# Plant-Derived Nanovesicles: A Comprehensive Review from Isolation to Clinical Translation—Unlocking Natural Nanocarriers for Biomedical Applications

**DOI:** 10.3390/biom16050705

**Published:** 2026-05-11

**Authors:** Xinyan Wang, Chenchen Yuan, Rong Lu

**Affiliations:** Marine College, Shandong University, Weihai 264209, China

**Keywords:** plant-derived nanovesicles, surface engineering, nanodrug delivery systems, biomedical applications, clinical translation

## Abstract

Plant-derived nanovesicles (PDNVs) are a class of nanoscale vesicles derived from plant tissues; they are particles with a lipid bilayer and no ability to replicate autonomously. As a type of bioactive natural nanocarrier, they demonstrate immense potential for application in 21st-century nanomedicine, skincare and nutritional health, owing to their excellent biocompatibility, low immunogenicity and targeted delivery capabilities. However, the clinical translation of PDNVs still faces key bottlenecks, including low extraction efficiency, complex purification processes, and immature engineering modification techniques. Compared to the wealth of systematic reviews in the field of Mammalian Extracellular Vesicles (M-EVs), research on PDNVs still lacks a comprehensive exposition of its multifaceted research progress. This review endeavours to comprehensively summarise the shortcomings over the last 60 years regarding PDNV purification processes, research progress, composition and characterisation, engineering modifications, functional mechanisms, clinical translation and market regulation. It discusses the feasibility of innovative approaches such as AI deep learning technologies, interdisciplinary integration and cross-application, and outlines the latest frontiers in PDNV research. It provides comprehensive and reliable reference material for future research and application strategies regarding PDNVs, offering theoretical support and practical guidance to overcome barriers to their industrialisation. This will facilitate the transition from limited laboratory research to clinical application and drive technological innovation in the next generation of naturally derived nanomedicines.

## 1. Introduction

In 1967, Halperin et al. observed via transmission electron microscopy that multivesicular structures in carrot cells were capable of fusing with the plasma membrane, subsequently releasing secondary vesicles containing contents into the extracellular space [1]. Over the following 37 years, as research into animal exosomes progressed, similar vesicular secretion phenomena in plant cells began to be re-examined. In 2004, Tse et al. defined the pre-vacuolar compartment in plants as a multivesicular body (MVB), demonstrating that it releases vesicles from its lumen through fusion with the plasma membrane, thereby providing morphological evidence for the existence of plant exosomes [2]. In 2007, Huckelhoven et al. revealed evidence that MVBs mediate the secretion of exosome-like particles in plants [3]. In 2008, Laura de la Canal et al. obtained extracellular fluid from sunflower seeds and detected the presence of phospholipid components [4]; the following year, they successfully isolated nanovesicles from sunflower seeds for the first time using a vacuum infiltration-centrifugation procedure [5]. Since 2013, an increasing number of researchers have identified PDNVs in various parts of a wide range of plants [6,7], and have conducted in-depth investigations into the isolation, characterisation, functions and mechanisms of these PDNVs [8]. Engineering modifications, clinical applications, industrialisation and cross-disciplinary applications have become cutting-edge focal points in the field of PDNVs.

At present, there is no standardised terminology for PDNVs. PDEVs (Plant-Derived Extracellular Vesicles) is a commonly used abbreviation in general research; however, strictly speaking, PDEVs must be obtained from the wash supernatant of plastids or from cell culture supernatants. PELNs (Plant-Derived Exosome-Like Nanoparticles) specifically refer to a subgroup of exosome-like nanoparticles, whereas vesicle-like particles obtained through methods such as juicing or homogenisation essentially belong to the category of Plant-Derived Nanoparticles (PDNPs). These constitute heterogeneous mixtures produced by tissue disruption, rather than vesicles actively secreted by cells. Due to the extremely low yield of PDEVs, most current applied research still focuses primarily on PDNPs. Furthermore, between 2009 and 2020, both vesicles actively secreted by living cells and particles generated by tissue disruption were often indiscriminately referred to as ‘PDEVs’. To balance academic rigour with practical application scenarios, this paper uniformly adopts the neutral generic term ‘PDNVs’ to encompass all types of plant-derived vesicle-like particles. In subsequent sections of this paper, clear distinctions will be made when the discussion differs due to variations in terminology; otherwise, the term “xxx (plant name)-derived nanovesicles” will be used.

PDNVs can be classified according to plant origin into edible, food-medicine dual-use, medicinal, and other categories [9,10,11]. The volume, composition, and physicochemical properties of PDNVs are heterogeneous, depending on cell origin, state, and environmental conditions.

**Figure 1:**
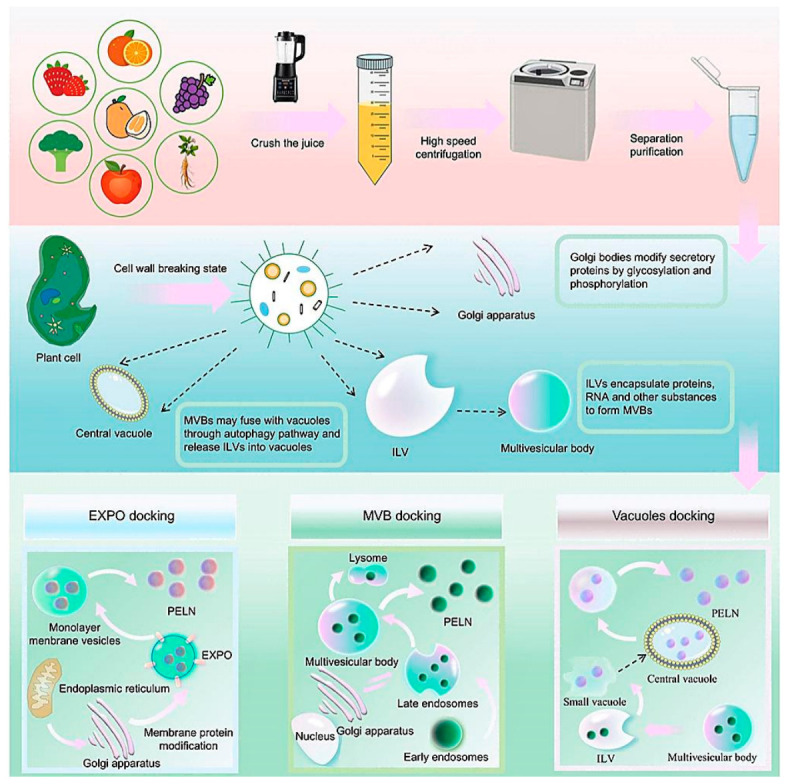
Schematic illustration of PDNV biogenesis pathways in plants: MVB-dependent secretion of TET8+ vesicles, EXPO-mediated direct release, and vacuole–plasma membrane fusion under biotic stress. This figure is reproduced from ref. [21], where these vesicles are referred to as PLNs (plant-derived exosome-like nanoparticles).

The current consensus regarding the biogenesis pathways of PDEVs includes the MVB pathway, the vacuolar pathway and the Exocyst-Positive Organelle (EXPO) pathway [12] (Figure 1). Among these, the MVB pathway has been studied in greater depth: endosomal invagination forms intraluminal vesicles, which are released into the extracellular space following fusion of the MVBs with the plasma membrane. This process involves a cascade of ESCRT complexes [13]; Furthermore, ESCRT-independent mechanisms exist in plants; for instance,FYVE domain protein required for endosomal sorting 1 (FREE1) forms biomolecular condensates via phase separation, which drives MVB membrane bending and scission independently of other ESCRT subunits and without ATP consumption [14]. In addition, AuTophaGy-related protein 8 (ATG8ylation)-mediated vacuolar membrane invagination has also been demonstrated to occur in a non-canonical manner [15]. The vacuolar pathway involves the fusion of vacuoles with the plasma membrane to release defensive vesicles [12]. The EXPO pathway is a plant-specific secretion pathway mediated by double-membrane organelles, marked by exocyst subunit EXO70 family protein E2(Exo70E2), and is considered distinct from the MVB-ESCRT system [16]. However, the consensus regarding these pathways is primarily based on qualitative descriptions, and quantitative studies on the relative contributions of each pathway across different plant species, tissue types and environmental conditions are still lacking. Furthermore, ESCRT-0 has not yet been definitively identified in plants [17], and there remains room for debate as to whether the EXPO pathway constitutes an independent extracellular vesicle (EV) secretory pathway [18]. It should be emphasised that most current isolation techniques are unable to effectively distinguish PDEV subtypes based on their biogenesis pathways. Although candidate markers such as tetraspanin 8 (TET8) have been proposed, there is a lack of widely validated universal markers. The International Society for Extracellular Vesicles (ISEV) recommends in the Minimal Information for Studies of Extracellular Vesicles 2023 (MISEV2023) guidelines that the direct use of biogenesis-based terminology be avoided unless subtypes have been clearly isolated and characterised [19].

PDNPs, on the other hand, are heterogeneous populations of nanoparticles formed passively during the mechanical disruption of plant tissue [12]. When plant material undergoes juicing, homogenisation or grinding, the contents released from cell lysis spontaneously assemble to form PDNPs, whose components include nanoscale lipid vesicles, fragmented membrane fragments, lipoprotein complexes and metabolite-enriched nanoparticles, and may also contain small amounts of co-separated PDEVs. Consequently, the composition of PDNPs directly reflects the lipid and metabolite profile of the source tissue, rather than being the result of selective loading. It should be emphasised that PDNPs should not be regarded as biological entities with a specific biological pathway, but rather understood as natural nanomaterials obtained following the processing of plant tissue [20].

Due to their natural origin and nanoscale dimensions, PDNVs have generally demonstrated low cytotoxicity and good biocompatibility in existing studies [22], and it has been confirmed that PDNVs from certain sources possess significant potential as tissue-targeted delivery vehicles [22]. This targeting ability is closely related to their unique membrane structure. The abundance of non-lamellar lipids and the asymmetric lipid bilayer structure confer a favourable membrane curvature on the vesicles, endowing them with an innate ability to penetrate biological barriers (such as the blood–brain barrier and plasma membranes) via lipid fusion [23]. However, the application of PDNVs still faces key challenges. Although their lipid bilayer structure provides some protection for the internal bioactive molecules, resulting in greater stability in the gastrointestinal environment compared to free nucleic acids or drug molecules, studies indicate that only approximately 10% of both plant-derived and mammalian-derived nanovesicles survive and are potentially absorbed by the intestine during in vitro simulated digestion [24,25]. This limitation suggests that to fully realise the immense potential of PDNVs in the biomedical field, numerous bottlenecks must still be overcome in the transition from laboratory research to clinical application.

**Figure 2:**
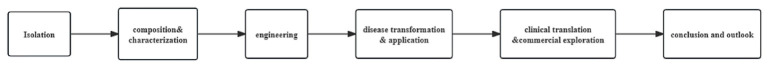
The review framework.

How can an efficient and reproducible purification process be established to obtain high-purity vesicles? How can their complex composition and structural characteristics be analysed using multi-dimensional characterisation techniques? How can engineered modifications be employed to further enhance their stability, targeting ability and drug-loading capacity? What preservation techniques should be selected to maximise the retention of PDNVs’ biological activity? How should the drug-loading efficiency of engineered PDNVs be assessed? How can their functional cellular mechanisms be elucidated and administration routes optimised to maximise therapeutic efficacy? What is the current status of clinical translation, market approval and regulatory oversight? Furthermore, how does research on PDNVs contribute to the modernisation of traditional Chinese medicine? An in-depth analysis of these key issues will directly advance the clinical translation of PDNVs, laying the groundwork for their transition from basic research to industrial application. The subsequent sections of this review will address these questions in turn (Figure 2), summarising current progress and exploring potential solutions.

## 2. Isolation

### 2.1. Preprocessing

PDNPs are typically extracted using tissue disruption methods. The plant material is thoroughly rinsed with tap water to remove coarse contaminants such as soil and dust, followed by thorough washing with distilled or deionised water to eliminate residual impurities and ions [26]. For rhizome materials such as ginger, the surface may be brushed with a soft-bristled brush to remove stubborn stains, taking care not to damage the epidermis. After washing, allow the material to drain naturally or gently blot the surface dry with absorbent paper. If used for aseptic experiments or cell culture, a surface disinfection step may be added, involving immersion in 70% ethanol for 30–60 s, followed immediately by rinsing with sterile water. The post-washing treatment depends on the type of material: succulent tissues (such as fruits and tubers) can be juiced directly; low-juice tissues (such as rhizomes and seeds) require prior soaking in a buffer (e.g., 2-(N-morpholino)ethanesulfonic acid (MES) buffer) followed by extraction via grinding or homogenisation [27,28,29,30,31]. Tissue disruption methods physically break down plant tissue to obtain vesicle-rich juice and are the preferred method for the large-scale extraction of PDNPs from tissues such as fruits and leaves [32,33,34]. However, whilst this method increases yield, it may introduce contaminants such as organelle membranes and plasma membrane fragments. Consequently, researchers have proposed various optimisation strategies, such as adding Tris-HCl during citrus juice extraction to eliminate pectin interference [35], or subjecting leaves to pectinase and cellulase digestion to remove cell walls [36].

The extraction of PDEVs requires the collection of naturally secreted vesicles from living vascular plant tissues, and the tissue infiltration method should be employed. Plant materials must undergo rigorous surface disinfection to prevent microbial contamination [37]. A buffer solution (e.g., Phosphate-Buffered Saline [PBS], MES) is introduced into the intercellular spaces via negative pressure permeabilisation and infiltration to collect apoplastic washing fluid (AWF). This method yields PDEVs of higher purity [31,38,39], but has the following limitations: the diluting effect of AWF results in lower product concentrations; the application of vacuum may cause minor cell damage, introducing a small amount of intracellular vesicle contamination [40]; vacuum permeabilisation may cause transient hypoxia in plant cells, inducing stress responses and altering the original metabolite profile of the apoplastic fluid [40]. In recent years, the enzyme method (macerozyme-mediated AWF collection) has emerged as an optimised alternative, enabling higher yields of PDEVs in a shorter time, with purity comparable to traditional methods, and is suitable for both mature leaves and seedling samples [41].

This study compared 19 strategies for isolating PDNVs by evaluating key indicators such as the level of evidence supporting each method, the trade-off between yield and purity, and scalability (Supplementary Table S1), with the aim of providing guidance on method selection for different research objectives.

### 2.2. Workhorse Methods

#### 2.2.1. Crude Extraction

Differential ultracentrifugation (dUC), differential centrifugation (DC) and precipitation are commonly used separation strategies for the coarse fractionation and preliminary enrichment of PDNVs. DC separates particles based on differences in their sedimentation rates; by progressively increasing the centrifugal force, particles of different sizes are precipitated in sequence. dUC utilises high centrifugal forces (typically ≥100,000× *g*) to precipitate nanoscale vesicles; Precipitation, primarily represented by the polyethylene glycol (PEG) precipitation method, utilises the molecular crowding effect of PEG to reduce the hydration of vesicles, thereby inducing their precipitation. All three methods offer the advantages of simple operation, high throughput and low cost, making them suitable for the rapid enrichment of PDNVs from large-volume plant samples; they are frequently employed as pre-treatment steps [42].

However, the separation mechanisms underlying these three methods mean they all share the common limitation of limited purity, albeit with different underlying causes. DC and dUC have limited resolution and cannot precisely separate components of similar sizes; the products are often contaminated with protein aggregates and particulate impurities of similar dimensions. Precipitation, on the other hand, lacks selectivity, causing all nanoscale particles to precipitate together; in addition to protein aggregates, the product also contains residual polymer (PEG), which may interfere with downstream analyses that are relatively sensitive to polymer residues [43,44,45]. Consequently, all three methods must be coupled with purification techniques such as density gradient centrifugation (DGC), size-exclusion chromatography (SEC) or ultrafiltration (UF) to obtain high-purity PDNVs [1,46,47]. Furthermore, during dUC and DC processes, the mechanical stress generated by high-speed centrifugation can easily cause deformation, rupture and aggregation of the vesicle structure, and prolonged centrifugation may affect vesicle integrity [48].

**Figure 3:**
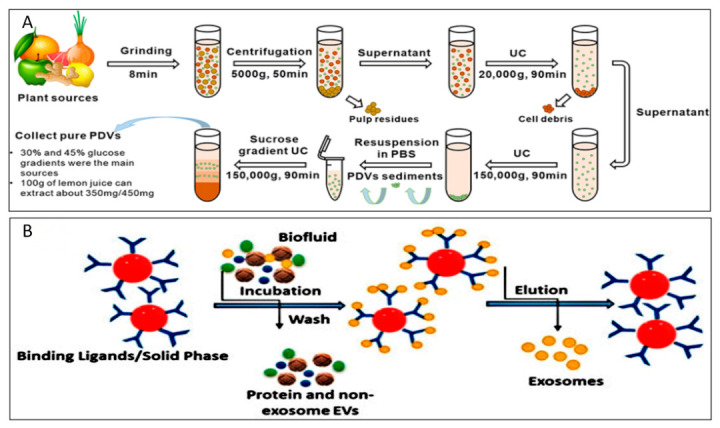
(**A**) Schematic diagram of PDEVs separation by ultrafiltration. (**A**) is reproduced from ref. [6]. (**B**) The foundational concept of the immunoaffinity-based approach for the extraction of exosomes. (**B**) is reproduced from ref. [45].

Each of these three methods has its own specific focus in practical applications. DC effectively removes large particulate impurities such as cell debris and fibres, whilst dUC is primarily responsible for precipitating nanoscale vesicles and is typically employed as a subsequent step following DC [49,50,51]. Furthermore, as UC (Figure 3A) is generally more costly, the PEG precipitation method is regarded as a low-cost alternative to UC. Studies have shown that the PEG6000 precipitation method can be used to isolate nanovesicles from ginger, yielding 60–90% of the output achieved by UC, with comparable product properties [52]. Zhang et al. used this strategy to isolate nanovesicles from tartary buckwheat, which exhibited well-preserved vesicular structure and biological activity, achieving a yield of 1.7795 g/kg under optimized conditions. Compared with vesicles obtained by UC (141.8 nm) from their previous study, the PEG-derived products showed a larger mean particle size (182.8 nm) [53,54].

#### 2.2.2. Purification

Following crude extraction and preliminary enrichment, further purification is required to obtain high-purity samples. Density gradient centrifugation (DGC), gradient ultracentrifugation (GUC) and SEC are commonly used purification strategies, but they differ significantly in terms of separation principles, sample integrity and suitability for specific applications.

Both GUC and conventional DGC utilise differences in particle buoyancy density for separation. GUC, as a mode of DGC, significantly improving purity; however, it is complex to operate, time-consuming (often requiring overnight processing), has a low recovery rate, and is difficult to scale up for production. It is suitable for mechanistic studies and downstream omics analyses requiring high purity and subpopulation separation [42,55]. Conventional DGC utilises media such as caesium chloride, sucrose or polydextrose to significantly improve the purity of DC crude extracts; however, it is prone to introducing exogenous substances, is time-consuming and has low recovery rates [28,30,46,56,57].

Sucrose density gradient centrifugation (SDGC) is a specific implementation of DGC [42]. The sucrose cushion double-stage ultracentrifugation method, developed on this basis, employs a two-step strategy: first, UC through 27% and 68% sucrose cushions to enrich vesicles between the two cushion layers, thereby removing large particulate impurities and reducing mechanical damage; followed by UC through an 8% to 60% sucrose gradient to purify the target vesicles. This method retains the high-purity advantage of SDGC whilst significantly improving yield and vesicle integrity by avoiding repeated precipitation [29].

SEC is a gentle technique that effectively removes small-molecule impurities and free proteins, yielding high-purity, structurally intact nanovesicles suitable for downstream applications requiring the preservation of vesicle activity. However, it has limitations such as long run times, high column costs, the need for sample dilution, and the potential for co-separation due to size overlap between lipoproteins and vesicles [31,58,59,60]. SEC is suitable for mechanistic studies and omics analyses requiring high purity and the preservation of vesicle integrity [47,61].

Both DGC and SEC can achieve high purity, but they have different focuses; researchers should weigh their options based on the objectives of downstream applications.

Combining UC with DGC or SEC can be considered an effective strategy for improving the purity of PDNVs. Kim et al. used a sucrose pad and sucrose gradient double-stage ultracentrifugation method to isolate high-yield, high-purity, and morphologically uniform ginseng-derived nanovesicles [29]. Bokka et al., on the other hand, first used dUC to preliminarily enrich micron- and nanoscale tomato-derived nanovesicles, followed by purification via a size-exclusion chromatography column packed with Sepharose CL-2B, achieving a high yield of 2.6 × 10^15^ particles per kilogram of fruit, which was significantly superior to density gradient ultracentrifugation [47].

#### 2.2.3. Scalable Production

Ultrafiltration (UF) and tangential flow filtration (TFF) are the two mainstream separation methods used to achieve large-scale production [55]. TFF is a mode of operation within UF and is widely regarded as the most promising route to large-scale production [62]; it has been successfully applied to the separation of nanovesicles from various plant sources, including ginger, broccoli, grapefruit and lotus leaves [63,64,65]. The two methods are fundamentally similar in their separation principles [49,62], with the distinction lying in their hydrodynamic design: UF employs dead-end filtration, which is simple to operate and offers rapid processing speeds (not exceeding 120 min per run), but suffers from significant membrane fouling issues; TFF utilises a flow pattern where the sample solution flows parallel to the membrane surface, effectively reducing the accumulation of contaminants on the membrane surface and making it more suitable for continuous production [62,63,64,65,66]. Both methods can process large-volume samples and operate under mild conditions, effectively preserving vesicle structure and biological activity; costs are relatively manageable, but both face limitations such as membrane pore blockage and the co-separation of particles of similar size, such as lipoproteins [62,63,64,65,66,67]; They can serve as pre-treatment steps for large-scale production, but typically require coupling with other techniques (such as SEC or anion exchange chromatography) to form a key component of a multi-step purification process, thereby further enhancing product purity [49,62].

When used in isolation, UF and TFF offer limited purity and are therefore often combined with SEC, anion exchange chromatography (AEX) or UC. Different combinations vary significantly in terms of throughput, purity and suitability for specific applications, and the choice should be based on the research objectives.

With respect to throughput and scalability, although the UF-SEC combination can significantly reduce protein contamination and maintain the integrity of vesicle morphology [27], it has low throughput and is difficult to scale up, making it suitable only for laboratory-scale preparation. In contrast, the Ultrafiltration–Anion Exchange–Fast Protein Liquid Chromatography (UF-AEX-FPLC) combination represents a scalable alternative. Zanotti et al. successfully isolated nanovesicles from broccoli seedlings [68], and the results confirmed that this method offers advantages in purity over conventional UC, whilst maintaining high recovery rates and product integrity [68,69]. However, it should be noted that this study did not include parallel controls using other separation methods, and thus its superiority lacks direct comparison; furthermore, as AEX relies on the surface charge of the vesicles, charge heterogeneity across different plant sources may affect separation efficiency [68]. The TFF-SEC combination offers both high throughput and scalability. TFF enables rapid concentration and removal of macromolecular contaminants, whilst SEC further removes soluble proteins. The combination of the two significantly improves purity and avoids mechanical damage to vesicles caused by UC, making it more suitable for industrial production requirements [70]. The continuous UF/DF method based on TFF principles also offers high throughput and scalability, achieving a recovery rate of 89.4% and an impurity removal rate of 99.7% when processing lemon juice [62]. In contrast, although the dual-cycle TFF (dcTFF) system can separate 30–200 nm vesicles in a single step [71], its low flux (in the μL/min range) and limited scalability make it more suitable for precise laboratory-scale separation rather than large-scale production [71]. The combination of TFF and UC has been applied in the separation of *Aloe vera-*derived nanovesicles, but UC itself is difficult to scale up [72].

In terms of product purity, significant differences exist between different combined strategies. The UF-SEC combination achieved a purity of 10 × 10^9^ particles/μg protein in cabbage, significantly outperforming PEG precipitation (0.242 × 10^9^) and ultracentrifugation (0.432 × 10^9^) [27]. The UF-AEX-FPLC combination was used to isolate PDNVs from broccoli, achieving a purity index of 1.0 × 10^9^ particles/μg protein. However, as this study lacked parallel controls (e.g., UC or SEC) under identical conditions, the relative performance of UF-AEX-FPLC compared to other methods remains to be systematically validated [68]. The TFF-SEC combination can significantly remove protein contamination and improve purity, whilst avoiding the mechanical damage to vesicles caused by UC, making it more suitable for industrial production requirements [70]. When treating lemon juice using the UF/DF continuous method, the relative EV content was 40.4%, slightly lower than the 44.3% achieved by SEC, whilst UC yielded only 5.6% [62]. Compared with the ExoQuick precipitation method, dcTFF increased the CD63 positivity rate by 1.3-fold and the CD63/Annexin V ratio by 70-fold, indicating higher PDNV purity and lower microvesicle contamination in the product [71]. TFF-AEX coupling may enable discrimination of functional subpopulations, yet its efficacy likely depends on batch-to-batch consistency of PDNV surface charge [73]. The combination of TFF and UC enables size sorting, concentration, and initial purification of vesicles; however, the UC step poses a risk of co-precipitation of impurities and suffers from low throughput, which limits large-scale production [72].

Recovery rates also differ, the continuous UF/DF method based on the TFF principle performs best. Giancaterino et al. achieved a recovery rate of 89.4% and an impurity removal rate of 99.7% when separating lemon-derived nanovesicles; however, the purity was slightly lower than that achieved by SEC, and the process relied on multi-step centrifugation pretreatment to control membrane fouling [62]. The dual-cycle TFF (dcTFF) system employs a series of 200 nm and 30 nm membranes to prevent membrane clogging, enabling the one-step separation of 30–200 nm vesicles [71]. Compared with the ExoQuick precipitation method, the separated products exhibit a richer profile of markers and higher biological activity [63,64,65]. The UC-TFF combination strategy leverages the complementary advantages of UC and TFF; however, it suffers from limitations such as process complexity, a lack of parallel controls, membrane fouling, and restricted product purity [72]. As its advantages are not significant, this approach is currently rarely employed.

Overall, TFF-SEC achieves a favorable balance among flux, purity, and recovery rate, and its effectiveness has been confirmed by multiple independent studies [70].

### 2.3. Promising but Need Further Validation

#### 2.3.1. Rapid Analysis and Microscale Preparation

Gel electrophoresis and Electrophoretic Liquid Density (ELD) both fall within the category of electrophoretic separation, but their principles and applications differ significantly. Gel electrophoresis is simple to perform and suitable for PDNV analysis and rapid purification [74], although electric field-induced heating may affect sample stability. ELD is a novel preparative technique combining electrophoresis and dialysis, which uses an electric field to drive charged small-molecule impurities (proteins, RNA) through a semi-permeable membrane towards the electrodes, whilst larger PDNVs are retained within the dialysis bag. This method is time-saving and convenient [75,76], yielding high-purity, structurally intact and bioactive PDNVs with yields comparable to those of UC. It has been successfully applied to the separation of various PDNVs [77,78] and is suitable for the rapid laboratory production of high-purity, structurally intact PDNVs. Despite limitations such as membrane pore blockage, restricted high-throughput application, and limited ability to separate impurities with similar properties, ELD’s manual buffer replacement and field reversal every 30 min could be improved through automation [77].

The capillary-channeled polymer (C-CP) fiber spin-down tip approach uses salt-enhanced hydrophobic interaction chromatography to efficiently separate nanovesicles while preserving their structural integrity. This low-cost, simple method is suitable for rapid separation of micro-volume samples (100 μL) and offers some potential for scaling up [79]. Jackson et al. [79] employed this method to isolate nanovesicles from 20 common fruits and vegetables. Evaluation based on the EV/protein ratio demonstrated that their purity was 10 times higher than that of UC and PEG [79]. However, mechanical homogenization during sample pretreatment may co-extract intracellular vesicles or bacterial outer membrane vesicles, compromising product purity. Moreover, current evidence comes primarily from a single study [79].

The kit-based extraction method is simple to operate and time-efficient, yielding PDNVs of high purity and good stability [28]; however, it is relatively costly and unsuitable for large-scale isolation, and polymer precipitation may introduce impurities that interfere with downstream analyses such as mass spectrometry. Commercially available PDNV isolation kits are primarily categorised into four types: polymer precipitation, column chromatography, magnetic bead-based methods, and tissue-specific kits. Among these, the ExoQuick kit utilises the molecular crowding effect of PEG [28,80,81,82] to reduce the hydration of PDNVs, causing them to precipitate. The isolated PDNVs retain structural integrity and yield higher than that of UC, but purity is lower [28]. Jang et al. combined ExoQuick with UF, further increasing product purity to 83.3%, but the yield was only approximately 24% of that achieved with ExoQuick alone [28]. The Exo-spin™ kit combines SEC with polymer precipitation. Its advantages and limitations are similar to those of ExoQuick. Notably, it may not effectively separate PDNVs from lipoproteins, leading to residual lipoprotein impurities. This can interfere with downstream proteomics or lipidomics analyses, as evidenced by the marked discrepancy between NTA particle counts and exosomal protein abundance [83].

Briefly, gel electrophoresis is the most economical choice for rapid PDNV detection. For higher purity from small sample volumes, C-CP Tip offers simplicity, whereas ELD provides better-validated purity. Commercial kits are suitable when convenience and low cost are prioritized. Notably, these methods still have limitations in automation or throughput, making it difficult to fully meet the demands of large-scale testing.

#### 2.3.2. High-Purity Preparation

Depending on the carrier to which the antibody is immobilised and the separation method employed, Immunoaffinity Capture (IA) (Figure 3B) is classified into three categories: Immunoaffinity Chromatography (IAC), Immunomagnetic Separation (IMS) and Enzyme-Linked Immunosorbent Assay (ELISA); these are often used in conjunction with microfluidics to enhance separation efficiency [84]. For the isolation of PDNVs, IMS is the predominant method, whilst ELISA is mostly used for post-isolation detection and validation; reports on the use of IAC for PDNVs are relatively scarce. IMS involves conjugating antibodies to magnetic nanoparticles to capture target vesicles through specific binding, followed by separation using a magnetic field; this method is rapid and easily automated [85,86]. In *Arabidopsis* research, TET8-antibody-conjugated magnetic beads have been successfully employed to isolate TET8-positive nanovesicles from vesicle washings [87]. However, this method faces challenges such as high costs associated with antibody conjugation and non-specific adsorption by the magnetic beads. Furthermore, the lack of universal conserved surface markers on PDNVs restricts isolation to specific subsets, such as TET8-positive nanovesicles [45,88]. Although ELISA enables the separation and quantitative detection of PDNVs with high sensitivity, it suffers from low throughput and susceptibility to interference from plant sample matrices [89]; consequently, it is primarily used for post-separation validation rather than as a primary separation method [90,91].

To overcome the bottleneck of marker deficiency, the design of the ExoSIC microfluidic chip [92] could be adapted, though this would require the development of plant-specific ligands and the optimisation of chip parameters and magnetic field strength. The integration of microfluidics with immunocapture represents a key direction for development in this field [84,86,93]. Future research could explore multi-ligand synergistic strategies, such as utilising a cellulose-based carrier surface where lectins recognise sugar chains, lipid-binding peptides anchor membrane structures, and metabolite receptors bind functional molecules; the synergistic action of these three components is expected to overcome the limitation of weak binding affinity associated with single ligands. IA enables the production of ultra-pure PDNV formulations [45,87,90,91,94]; however, this strategy currently remains highly dependent on knowledge of PDNV surface markers, and systematic research is urgently required.

#### 2.3.3. Large-Scale Preparation

AEX has traditionally been used primarily for the separation and purification of biomacromolecules and for the analysis and detection of inorganic and organic anions. When applied to the separation of PDNVs, specific modifications are required to account for the characteristics of the vesicles [68,69,95]. As mentioned in Section 2.2.3 ‘Scalable Production’, Zanotti et al. established a purification platform combining TFF/UF with AE–FPLC. After concentration and buffer exchange by TFF/UF, the sample was treated with Micrococcus nuclease to remove free nucleic acids that compete with PDNVs for binding. To avoid the low mass transfer and pore blockage issues of conventional porous columns, they selected the CIMmultus™ EV monolithic column, whose open channels better accommodate nanovesicles [68].

The Aqueous Two-Phase System (ATPS) is suitable for the preliminary purification and concentration of PDNVs from complex plant samples. This method employs mild separation conditions, effectively maintains vesicle structural integrity, and is cost-effective, whilst simultaneously and efficiently removing impurities such as proteins, fatty acids, phenol red and plant secondary metabolites [6,42,96]. It has currently been successfully applied to the separation of PDNVs from various plant lysates, including pomegranate, celery root, ginger and turmeric [97,98,99] (CN119242556A). However, ATPS has limitations. Phase composition (e.g., PEG molecular weight, PEG/Dextran(DEX) ratio must be optimized per sample, and variations in pH and impurities across sources further complicate this process [96,97,100]. Furthermore, residual dextran in the final product may interfere with downstream analyses such as Western blotting and RNA extraction, and increase solution viscosity [96,97,100]; this can be addressed by setting up a dextran blank control or by coupling the method with SEC. A study on human bone marrow mesenchymal stem cell (BM-MSC)-derived EVs found that although ATPS effectively removes free proteins and achieves a high particle-to-protein ratio, its functional purity (i.e., the proportion of tetraspanin-positive EVs in the final product) remains significantly lower than that of SEC-based methods [101]. Therefore, ATPS is more suitable as an initial purification and concentration step for complex samples, rather than as a final method for preparing ultra-high-purity PDNVs. In the future, the phase composition and washing conditions could be further optimised to enhance separation selectivity [97,100].

The super-absorbent polymer beads method (SAP) utilises polymeric nanochannels to selectively absorb water and small-molecule impurities (including salt ions, DNA, RNA and small-molecule proteins), whilst larger nanovesicles (50–1000 nm) are excluded, thereby achieving highly efficient concentration. However, some nanovesicles may be lost due to channel size limitations. Taking the separation and purification of nanovesicles derived from *Perilla* leaves as an example [102], the concentration of nanovesicles can be increased by 8 to 9 times after SAP treatment. This method comprises two strategies: SAP(C) and SAP(S); the former yields a higher output and takes less time, whilst the latter offers higher purity and allows for buffer replacement; SAP(S) is therefore recommended as the preferred choice. After subsequent SEC purification, the overall yield was approximately 26%. Compared to PEG precipitation, this method avoids polymer contamination and achieves higher purity; compared to ultrafiltration (UF), it offers comparable purity with superior recovery [102]. The entire process takes only a few hours, with low cost per sample, no need for an ultracentrifuge, simple operation, and preservation of the structural integrity and biological activity of the nanovesicles. SAP is suitable as a low-cost pretreatment step for plant sap nanovesicles, but must be combined with SEC to obtain high-purity products; furthermore, excessively high sample viscosity reduces absorption efficiency.

### 2.4. Emerging but Unvalidated

**Figure 4:**
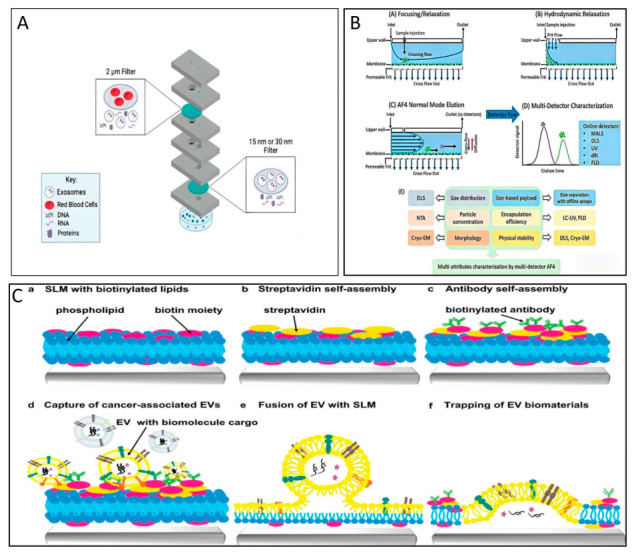
(**A**) Diagram illustrating the operating principle of the microfluidic device: this innovative microfluidic system employs a dual filtration method. (**A**) is reproduced from ref. [106] (**B**) Representative diagram of relaxation and elution process in AF_4_. (**B**) is reproduced from ref. [115]. (**C**) Scheme of extracellular vesicle (EV) capture by supporting lipid membranes (SLM). (**C**) is reproduced from ref. [104].

Microfluidics (Figure 4A), Asymmetric Flow Field-Flow Fractionation (AF_4_) (Figure 4B), Molecularly Imprinted Polymer (MIP) and Lipid Microarray (Figure 4C) have not yet been fully established for the isolation of PDNVs, although their levels of maturity vary; their principles and performance offer potential tools for advanced research into PDNVs.

AF4 has been successfully applied in the separation of M-EVs, providing valuable technical insights for research on PDNVs [100,103,104]. AF_4_ utilises differences in particle diffusion rates under cross-flow conditions to achieve size-based separation; it is label-free, involves low shear stress, and offers high size resolution [100]. Although the lipid microarray platform demonstrates promising performance in the isolation of mammalian extracellular vesicles (M-EVs), it remains at an early stage of technological development. The platform enables efficient EV capture via antibody recognition of surface markers, achieving a sensitivity of 5 × 10^3^ EVs/mL with sample volumes as low as 30–50 μL. Notably, the method does not require ultracentrifugation; complex samples can be directly loaded after simple filtration. Furthermore, the platform preserves EV cargo integrity, allowing for downstream analyses [104,105]. Both techniques are limited by low processing throughput (μL/min scale) and high equipment costs. AF4 suffers from co isolation of particles with similar hydrodynamic sizes, while the lipid microarray depends on specific antibodies, yet PDNVs lack universal surface markers [103,104,105,106].

MIP has demonstrated preliminary feasibility in M-EVs (with better enrichment efficiency than PEG precipitation), but has not yet been applied to PDNVs [94,107]. It offers advantages such as independence from biomarkers, excellent chemical stability, and low cost, yet faces challenges concerning template heterogeneity, biocompatibility, and scale-up production [107,108,109,110]. Microfluidics remains in the early stages of exploration for PDNV separation, with no reports of mature applications to date [111]. Its advantages include short processing times (some devices complete the process within 30 min), low sample consumption (microlitres), and the ability to achieve targeted enrichment of specific subtypes through integrated immunoaffinity [112,113]; however, it suffers from high equipment costs, low throughput, and a tendency to clog [112,113,114].

The aforementioned technologies are unlikely to replace traditional methods for PDNV separation; however, they offer unique value in high-value-added research such as PDNV subtype analysis, targeted enrichment of specific subpopulations, and drug carrier screening. AF_4_ and Lipid Microarray can directly provide technical insights for PDNV research, whilst Microfluidics and MIP are still in their early or preliminary stages and require further validation. The integration of PDNV-specific markers or the optimization of throughput design may further expand its future applications [88,104,105].

In summary, the choice of PDNV isolation method must be weighed against the requirements of downstream applications. For rapid assessment of the presence of PDNVs in a sample, C-CP Tip and gel electrophoresis may be employed. When specific subpopulations (such as TET8-positive) need to be obtained, immunoaffinity capture is a viable option, although it is limited by the lack of universal surface markers on PDNVs. For large-scale production intended for animal experiments, the combination of AEX-FPLC and ATPS-SEC is currently the most established approach. When maintaining vesicle integrity is required for functional studies, SEC or ELD is more suitable. Where low-cost concentration of plant sap is required, SAP or ATPS may be used as a pre-treatment step, but must be combined with SEC to meet purity requirements. For high-purity samples intended for omics analysis, sucrose pad double-stage ultracentrifugation or SEC may be considered. When processing large-volume samples, UF or TFF are the mainstream choices for large-scale production, but must be coupled with SEC or AEX to further enhance purity. For the crude extraction and preliminary enrichment stages, DC, dUC and PEG precipitation can all be used for rapid enrichment of large-volume samples, but must be coupled with a purification method. In summary, no single method is suitable for all scenarios; a multi-step combined strategy is typically the optimal solution for balancing yield and purity.

It should be noted that even when employing the same purification method, separation results may vary depending on the initial state of the sample. For example, when separating vesicles from *Portulaca oleracea* L. using sucrose gradient centrifugation, there are significant differences in results between fresh and dried materials [116]. Furthermore, there are significant variations in PDNV yields across different plant sources; for instance, yields per gram of tomato, cabbage and red cabbage were only 1.12 × 10^10^, 1.50 × 10^11^ and 1.10 × 10^11^ particles, respectively [27], making direct cross-species comparisons of yield of limited value. Furthermore, inconsistent yield units (mg/kg, particles/g, particles/mL, etc.) and varying detection methods (BCA, NTA, Qubit), along with considerable discrepancies in how different studies evaluate the same method, collectively constrain data comparability.

## 3. Composition and Characterization

PDEVs possesses an active, selective mechanism for sorting its contents [12] and represents a biological process by which plant cells regulate secretion. In contrast, PDNPs lacks such a sorting mechanism; its contents are the result of passive encapsulation during mechanical disruption [12] and merely reflect the lipid, metabolite and RNA profiles of the source tissue. This fundamental distinction determines their different functional and application-specific roles. Therefore, PDEVs are better suited than PDNPs for studying cross-kingdom communication and signal transduction.

### 3.1. Composition

**Figure 5:**
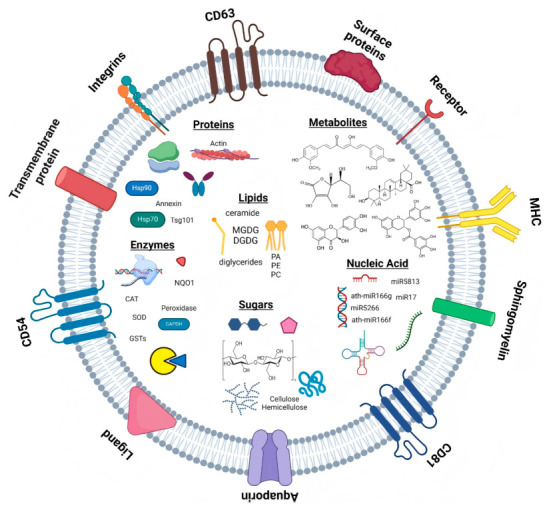
Structural characteristic of PDNVs. The figure is reproduced from ref. [117], where these vesicles are referred to as plant-derived exosome-like nanovesicles.

PDNVs are primarily composed of four major categories of components: lipids, proteins, nucleic acids and plant metabolites (Figure 5).

#### 3.1.1. Lipid

Common phospholipids found in PDNVs, such as phosphatidic acid (PA), phosphatidylethanolamine (PE) and phosphatidylcholine (PC), serve as basic markers for confirming the structural integrity of the vesicle membrane [118]. Furthermore, they are rich in plant-specific plastid membrane lipids, such as di-galactosyl diacylglycerol (DGDG) and mono-galactosyl diacylglycerol (MGDG) [75]. These glycolipids serve as the primary carriers of carbohydrates in PDNVs, providing key indicators for distinguishing PDNVs from M-EVs. Free carbohydrates (such as β-glucan) are present in limited varieties [119]. Meanwhile, cholesterol is present in extremely low concentrations in PDNVs—and is often undetectable—as it is not enriched [75,120].

Currently, most studies remain at the macroscopic level regarding the efficacy of lipid components. On the one hand, PA has been shown to possess distinct biological functions, such as directly binding to *Porphyromonas gingivalis* outer membrane proteins to inhibit their growth [121], and upregulating Foxa2 to improve insulin resistance in mice induced by a high-fat diet [122]; PC, meanwhile, is involved in maintaining the integrity of the colonic mucosal barrier in mice [123,124,125]. On the other hand, speculations regarding the functions of PE and PI are primarily based on their known functions in other biological systems (such as membrane fusion and signal transduction) [126,127], and direct evidence within PDNVs remains insufficient [126,128,129,130]. However, the failure to precisely identify the specific lipid molecules playing a central role has, to some extent, hindered an in-depth analysis of the lipid mechanisms underlying PDNVs.

Lipidomic studies indicate that the lipid profiles of different PDNVs vary significantly; these unique lipid profiles form the molecular basis for their selective cellular uptake [75,80,120,131,132]. For instance, nanovesicles derived from ginger and grapes are rich in PA, whilst PE and PC dominate in nanovesicles derived from grapefruit and garlic [75]; the lipid composition of orange-derived nanovesicles is similar to that of vesicles derived from grapefruit, which belongs to the same Rutaceae family, and their PE/PA ratio is the opposite of that found in vesicles derived from ginger or grapes [127]. Even when PDNVs from different plant sources are rich in a particular lipid, their target cells may differ entirely [7,133,134]. For example, grape-derived nanovesicles containing 53.2% PA are primarily taken up by intestinal target cells [7], whereas ginger-derived nanovesicles containing 37.0% PA are primarily taken up by liver cells [134], suggesting that the fine structure and composition of lipids may be more important than the mere content of a single lipid. Microbial interactions also exhibit lipid-specific preferences; for example, *Lactobacillus rhamnosus* prefers to take up PA-rich ginger-derived nanovesicles [75,135], whilst members of the Ruminococcaceae family preferentially endocytose PC-rich grapefruit-derived nanovesicles [75,134].

#### 3.1.2. Protein

Research into the protein composition of PDNVs faces challenges due to their low abundance, diverse nature, and the need to cross-reference various plant protein databases; consequently, the number of identified proteins remains relatively limited [136].

Cross-species comparative analyses reveal that PDNV proteins exhibit a high degree of functional conservation. The identified proteins primarily belong to three core biological processes: defence and stress responses (e.g., RPM1-interacting protein 4 and PENETRATION 3 in *Arabidopsis*-derived nanovesicles, and pathogenesis-related proteins in blueberry-derived nanovesicles [132,137]), vesicular transport and substance translocation (e.g., clathrin heavy chain, COPI/COPII coat proteins and patellin, which are enriched in citrus-derived nanovesicles, as well as annexins and aquaporins in tomato-derived nanovesicles [35,132]), and signal transduction and metabolic regulation (e.g., heat shock protein 70/80(HSP 70/80) , 14-3-3 proteins and antioxidant enzymes, which are highly expressed in nanovesicles across multiple species [132,137]).

Research into the signature proteins of PDNVs has largely established a clear hierarchy ranging from universal conserved markers to species-specific markers. At the basic identification level, PDNVs share conserved proteins with M-EVs, including tetraspanin homologs (e.g., CD9, CD63, CD81), ESCRT-related proteins (e.g., tumor susceptibility gene 101 protein [TSG101] and ALG-2-interacting protein X [ALIX]), and heat shock proteins (e.g., HSP70 and HSP90) [138,139,140], as well as pan-plant markers (such as PENETRATION1[PEN1], tetraspanin 8[TET8], and the patellin family), which have been validated across multiple species including *Arabidopsis*, *sorghum*, and apple [35,141,142,143]. In terms of characteristic differentiation, recent studies have proposed plant-specific marker families, including fasciclin-like arabinogalactan proteins (FLAs), germin-like proteins and patellins [35,144], among which glycosylphosphatidylinositol-anchored FLAs (e.g., FLA10 and FLA13) are regarded as promising plant-specific markers due to their high abundance and conservation [144]. Furthermore, the discovery of markers such as Exo70, aquaporins, Ras-related proteins in brain GTPases and coatomer subunits has provided key clues for further distinguishing PDNV subgroups and elucidating their biological pathways and functional specificity [35,132,142,145]. However, despite the growing number of candidate markers, the lack of standardised surface markers validated across a wide range of species remains a core bottleneck constraining the standardisation of isolation and characterisation protocols in this field [146].

#### 3.1.3. Nucleic Acid

PDNVs carry various nucleic acids (miRNAs, sRNAs, DNA, and other non-coding RNAs), among which miRNAs are the most extensively studied.

The stability of plant-derived miRNAs stems from 2′-O-methylation at the 3′-terminus, which inhibits degradation, and is also associated with sequence and high guanine-cytosine (GC) content [147,148,149]; however, stability varies among different miRNAs [150]. Some plant-derived miRNAs are resistant to harsh cooking processes such as boiling and steaming, and can withstand human saliva rich in ribonuclease, as they are protected by the food matrix and nanovesicles. PDNVs are mainly absorbed in the human gastrointestinal tract, particularly the intestine [151]. Plant miRNAs are protected by vesicle encapsulation and RNA-binding proteins (e.g., Argonaute 2) to partially resist enzymatic degradation [150], with translocation pathways including transporter-mediated transport, endocytosis, immune cell uptake, and epithelial paracellular diffusion [150].

Classified by mechanism of action, miRNAs derived from PDNVs primarily exert their biological functions through three modes: direct cross-kingdom regulation, indirect cross-kingdom regulation, and tissue protection and homeostasis maintenance.

Direct cross-cellular regulation refers to miRNAs entering pathogen cells to directly target their functional genes. For example, miR-396a-5p and rlcv-miR-rL1-28-3p carried by ginger-derived nanovesicles can target the *Nsp12* and *spike* genes of SARS-CoV-2, respectively, significantly inhibiting virus-induced cytopathic effects and acute lung injury [152]. *Houttuynia cordata*-derived nanovesicles deliver miR858a/b and miR166a-3p, which directly bind viral RNA to inhibit replication by targeting the *nucleoprotein (NP)* gene of H1N1 and the *ORF1ab* in SARS-CoV-2, respectively [151].

Cross-membrane regulation refers to miRNAs entering host cells to regulate inflammatory, metabolic or proliferation-related pathways. Regarding the regulation of inflammatory pathways, pab-miR396a-5p carried by *Perilla frutescens* leaf-derived nanovesicles inhibits the activation of the NF-κB and JAK-STAT pathways by targeting *HSP90* mRNA, thereby blocking the IL-17-driven inflammatory cascade and reducing the release of pro-inflammatory factors such as IL-17A, IL-6 and TNF-α [153]; miR-156e, miR-162 and miR-319d in blueberry-derived nanovesicles, respectively, target inflammation-related genes such as *prostaglandin I2 synthase* (*PTGIS)*, *mitogen-activated protein kinase 14* (*MAPK14)* and *phosphodiesterase 7A* (*PDE7A)*, downregulate TNF-α-induced pro-inflammatory gene expression, inhibit NF-κB pathway activation, and alleviate oxidative stress by enhancing the expression of antioxidant genes such as *heme oxygenase 1* (*HMOX1)* and *nuclear respiratory factor 1* (*NRF1)*, thereby protecting endothelial cells from damage [154]. In terms of metabolic pathway regulation, miR-375 in ginger-derived nanovesicles improves high-fat diet-induced insulin resistance by inhibiting *aryl hydrocarbon receptor*-mediated pathways [155]. In terms of the regulation of proliferation and apoptosis pathways, miRNAs in *Brucea javanica*-derived nanovesicles induce apoptosis in breast cancer cells by inhibiting the PI3K/Akt/mTOR pathway [156].

In terms of organizational protection and homeostasis maintenance, ata-miR156c-3p in nanovesicles derived from *Lycium ruthenicum* Murray protects rat adrenal pheochromocytoma cell line from Amyloid-beta-induced neurotoxicity [157]; miRNAs in grape-derived nanovesicles activate the *Wnt/TCF4* pathway in intestinal stem cells, promoting intestinal mucosal repair and maintenance of homeostasis [158]. miR-7972 in nanovesicles derived from *Rehmannia glutinosa* alleviates lipopolysaccharide-induced pulmonary inflammation by targeting the *GPR161*-mediated Hedgehog pathway; Additionally, it regulates gut microbiota composition by inhibiting the bacterial virulence gene *stx2* [159]. Furthermore, *Portulaca oleracea* L-derived nanovesicles protect the colonic epithelial barrier and increase the abundance of beneficial bacteria in a mouse model of DSS-induced colitis, whilst also specifically targeting inflamed tissues [160].

It should be noted that the aforementioned functional studies are primarily confined to cellular and animal models, and their relevance to human physiology remains unclear. The phenomenon of trans-membrane communication has been confirmed, and the selective loading mechanism mediated by RNA-binding proteins is gradually being elucidated [161], providing a theoretical basis for the engineering of specific functional PDNVs. Given their oral bioavailability and nucleic acid protection capabilities, PDNVs hold promise as ideal carriers for oral nucleotide drugs. However, significant gaps remain in current research; reports on sRNA, DNA and other non-coding RNAs are relatively scarce, and their biological functions and delivery mechanisms require further elucidation.

#### 3.1.4. Secondary Metabolites

Secondary metabolites are key mediators of plant defence and signal transduction, and some of these compounds can be encapsulated within the lipid bilayer or lumen of PDNVs [162]. PDNVs deliver these metabolites to mammalian cells via endocytosis [163], with components such as polyphenols, terpenoids and alkaloids exhibiting biological activities including anti-inflammatory, antioxidant and anti-tumour effects in cross-species regulation [164,165].

Polyphenols are among the most common secondary metabolites in PDNVs and can be further classified into flavonoids, phenolic acids and other phenolic compounds. Flavonoids such as naringin, naringenin and quercetin are commonly found in nanovesicles derived from sources such as grapes, grapefruit and Aloe vera [96,136,164,166,167,168]; anthocyanins have been reported in nanovesicles derived from blueberries and pomegranates [151,169]. Phenolic acids are also widely present, such as ferulic acid in nanovesicles derived from grapes and broccoli, chlorogenic acid in tea [170,171,172]. Furthermore, terpenoids (such as cannabidiol, cycloartenol, and ginsenoside Rg3) and alkaloids (such as tabersonine and vinpocetine) have also been identified in various PDNVs [34,36,159,173].

Terpenoids are widely distributed in plants, with citrus fruits being one of their primary sources [167]. For example, cannabis-derived nanovesicles contain the anti-cancer active ingredient cannabidiol (CBD) [173], ginseng-derived nanovesicles contain ginsenoside Rg3 [34]. 

Alkaloids are another important class of secondary metabolites, such as ephedrine and berberine. Alkaloids are widely distributed in the plant kingdom, with significant variations in their content and types across different plants. For example, *Catharanthus roseus*-derived nanovesicles contain various indole alkaloid components, such as vinpocetine [36], which is clinically used to treat cerebrovascular diseases and cognitive dysfunction.

Based on the available evidence, although PDNVs carry a variety of secondary metabolites, their loading is highly selective rather than passively incorporating all plant-derived components [174]. Studies have found that vitamin C and naringin, which are abundant in orange juice, are not detected in purified orange-derived nanovesicles. In contrast, the recognized anticancer components sulforaphane and indole-3-carbinol are present in purified broccoli-derived nanovesicles, albeit at extremely low concentrations [75]. These findings suggest that future research should focus on the actual loading levels of secondary metabolites in PDNVs and their biological contributions, avoiding the direct equating of the compositional profile of plant tissues with the cargo profile of PDNVs.

The aforementioned components possess anti-inflammatory, antioxidant or anti-cancer activity in traditional pharmacology, suggesting that the biological effects of PDNVs may partly stem from the secondary metabolites they carry. The diverse bioactive molecules carried by PDNVs are co-delivered to recipient cells in their natural state, potentially generating synergistic effects that amplify their biological impact. Although numerous studies have elucidated the mechanisms of action of PDNVs at the in vivo level, their intracellular fate at the subcellular level remains to be thoroughly elucidated, including key aspects such as the efficiency of escape from endosomes and the success rate of miRNA loading onto the RNA-induced Silencing Complex (RISC). At the same time, the precise identification and quantification of small-molecule metabolites within them is equally crucial [175]; this not only helps to reveal their specific contributions to synergistic effects but also lays the foundation for optimising the clinical application of PDNVs. Future research could systematically analyse the distribution patterns of metabolites by developing highly sensitive methods (such as liquid chromatography-tandem mass spectrometry), and combine molecular pathway analysis with the loading of specific metabolites to design multifunctional engineered PDNVs.

Notably, relatively high levels of secondary metabolites are frequently detected in PDNVs purified by SDGC. However, SDGC suffers from co-purification of non-target contaminants and is ineffective at removing small molecules from the sample. More critically, these co-purified contaminants include lipoproteins, protein aggregates, and polysaccharide fragments, which are either rich in or can adsorb secondary metabolites. Their densities largely overlap with those of the vesicles, making them inseparable by sucrose gradients. Consequently, it remains unclear whether the high levels of detected metabolites reflect genuine enrichment in vesicles or are overestimated due to contaminant co-purification. The key to resolving this issue lies in designing rigorous control experiments. For instance, in a study on olive-derived nanovesicles, repeated centrifugation washes were explicitly used to remove free phenolic compounds and reversibly bound contaminants, ultimately yielding a vesicle-associated phenolic content of 2.1–4.6 mg hydroxytyrosol equivalents per gram of raw material [176]. Future studies should incorporate additional washing steps, secondary purification by size-exclusion chromatography, and control experiments that remove free metabolites, thereby enabling accurate assessment of the true cargo of secondary metabolites in PDNVs.

### 3.2. Characterization

The identification and characterisation of PDNVs rely on a comprehensive analysis of key parameters such as their morphology, particle size, surface charge, surface markers and molecular composition.

Electron microscopy is the primary technique for morphological observation. Transmission electron microscopy (TEM) and scanning electron microscopy (SEM) provide images at nanometre resolution; however, samples must undergo dehydration and fixation, which can easily cause PDNVs to deform into a characteristic cup-like structure [75]. Cryo-electron microscopy (cryo-EM), however, bypasses the fixation and staining steps, revealing a more spherical, true morphology under near-physiological conditions. Atomic force microscopy (AFM) not only allows observation of surface morphology but also enables the measurement of PDNVs’ mechanical properties and the quantitative assessment of biomarkers [94,177].

Particle size and surface charge analysis are critical aspects of PDNV quality control, and the particle size and surface charge of PDNVs vary depending on the source species. Conventional dynamic light scattering (DLS) technology, due to its low resolution and inability to measure particle concentration, is gradually being replaced by nanoparticle tracking analysis (NTA). By tracking the Brownian motion of particles in the 10–2000 nm range, NTA can accurately calculate the hydrodynamic diameter and concentration of PDNVs; it offers higher resolution and more stable results, and has now become the preferred technique for particle size characterisation [75]. In addition, techniques such as laser transmission spectroscopy (LTS) are also applied in the quantification of PDNVs. Residual cell debris from the extraction process can significantly interfere with NTA results, so sample purity is crucial for data reliability.

Surface markers of PDNVs must be distinguished between the two categories: PDEVs and PDNPs. PDEVs possess well-defined protein markers; classic examples include TET8, PEN1 and Exo70E2, whilst recently identified candidates include Aquaporin and FLAs, which exhibit a degree of conservation across multiple species [12]. In contrast, as PDNPs are passively formed heterogeneous particles, they lack universal protein markers; their identification relies more heavily on their enriched lipid, metabolite and RNA profiles.

Fluorescence-labelled multi-parameter analysis platforms (such as nFCM and fluorescence NTA) rely on antibodies or fluorescent dyes to specifically recognise surface markers on PDNVs, enabling the quantitative detection and tracking of specific subpopulations; however, their application is limited by the availability of known markers [75,178]. Local surface plasmon resonance (LSPR) is a label-free technique that determines binding kinetic parameters and concentration by monitoring real-time changes in refractive index caused by ligand-target binding; it offers high sensitivity but has not yet been widely applied in the field of PDNVs [94,177]. Capillary electrophoresis (CE) does not rely on specific reagents; it assesses sample purity and heterogeneity based on differences in the electrophoretic mobility of PDNVs in an electric field. It can detect soluble contaminants and macromolecular aggregates; however, its nanolitre-scale sample volume means the method is only suitable for analytical characterisation and cannot readily replace high-throughput techniques such as NTA [179,180].

The identification and characterisation of PDNVs still face multiple challenges. Firstly, the lack of specific molecular markers remains a key bottleneck; although candidate markers such as PEN1 and TET8 have been proposed and validated in some species, they are still far from becoming a standardised ‘gold standard’ on a large scale [143,181]. Second, the marker systems established for mammalian EVs are not fully applicable to plants, as the conservation of homologs of proteins such as CD9 and HSP70 in plants remains unclear [76,146,163,182]. Thirdly, the lack of standardised isolation and purification methods leads to highly heterogeneous samples, with significant variations in product purity, integrity and particle size, making direct comparisons between studies difficult; for example, NTA is prone to interference from impurities and requires extremely high sample purity [75]. Finally, the biological effects of PDNVs are often the result of synergistic interactions between multiple components, making it difficult to attribute them precisely to any single specific component; there is a lack of standardised validation procedures to distinguish between primary and synergistic effects.

## 4. Engineering

The engineering of PDNVs involves modifying natural PDNVs through physical, chemical or biological means to enhance their targeting ability, drug-carrying capacity and stability, or to confer new therapeutic functions upon them, thereby increasing their potential for application in drug delivery and disease treatment.

### 4.1. Engineering Transformation Strategy

#### 4.1.1. Methods of Loading Substance

**Figure 6:**
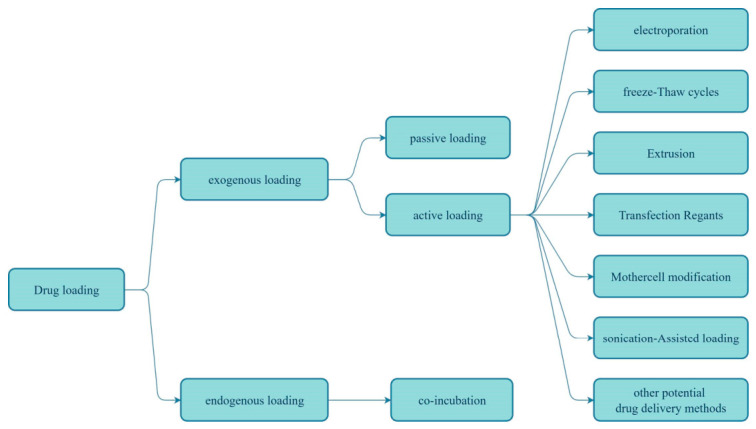
Drug loading methods.

Depending on the sequence of loading and PDNV separation, the methods by which PDNVs are loaded with drugs are classified into two categories: endogenous loading and exogenous loading (Figure 6).

##### Endogenous Loading

Endogenous loading involves co-incubating donor plant cells with the target drug prior to PDNV secretion, thereby allowing the drug to be naturally encapsulated during vesicle formation [183]. This method is particularly suitable for macromolecules such as proteins and nucleic acids; it enhances drug stability, reduces off-target toxicity, and, compared to exogenous methods such as electroporation, better preserves the integrity of the vesicles and their cargo. However, its application in the field of PDNVs still faces numerous challenges: loading efficiency is limited by the cells’ endocytic efficiency for the drug, and some cargo may be degraded by lysosomes; the physiological state of the donor cells affects drug loading capacity and vesicle yield, leading to significant batch-to-batch variability and posing difficulties for standardised production. Therefore, future efforts should combine approaches such as genetic engineering and optimisation of culture conditions to synergistically improve the efficiency and controllability of endogenous loading.

##### Exogenous Loading

- Passive loading methods

Passive loading methods, such as the incubation method, involve simply mixing PDNVs with a drug solution and incubating the mixture for a period at an appropriate temperature (which must take into account the properties of the drug, the characteristics of the PDNVs, the experimental objective and the conditions). The drug then spontaneously diffuses into the interior of the PDNVs. This method is simple to perform, requiring no complex equipment or special conditions, and has minimal impact on the structure and activity of the drug. However, the loading efficiency is relatively low, making it suitable for small-molecule, lipophilic drugs such as curcumin, doxorubicin and paclitaxel [184].

In vitro experiments have shown that cabbage-derived nanovesicles loaded with the chemotherapeutic drug DOX via incubation significantly inhibit the activity of human colon cancer SW480 cells [27,185]; lemon-derived nanovesicles efficiently loaded DOX via direct incubation. In vitro experiments showed that the drug-loaded vesicles retained their proliferation-inhibiting activity against cervical cancer HeLa cells, drug-resistant ovarian cancer cells and breast cancer cells, whilst significantly reducing toxicity to human embryonic kidney cells (HEK293T); Animal studies further confirmed that the drug-loaded vesicles reduce DOX accumulation in the heart [185,186,187]; Acerola-derived nanovesicles loaded with microRNA, when administered orally, inhibit the expression of target genes in the mouse intestine and liver. Cell experiments further revealed that this complex is resistant to degradation by nucleases and strong acids and bases, and exerts gene silencing functions through endocytosis [188].

Several chemical permeabilisation methods can be considered to enhance drug loading efficiency. Lu et al. leveraged the natural photosensitivity of *Pueraria lobata*-derived nanovesicles [189]. By triggering ROS-mediated lipid peroxidation via LED illumination, they achieved transient membrane permeabilization, resulting in an ~80% loading efficiency for FITC-dextran. The engineered vesicles maintained good stability for up to 30 days [189]. Kocholata et al. mixed nanovesicles derived from tobacco callus tissue with fluorescently labeled siRNA and added saponins (at a final concentration of 0.1 mg/ml in the mixture). After incubating the mixture at 37 °C for 1, 4, 6, or 24 h, free siRNA and saponins were removed through two rounds of UC (100,000× *g*, 1 h, 4 °C) [190]. However, the cholesterol content in PDNV membranes is typically very low, whereas the core mechanism of the saponin treatment method relies on saponin molecules specifically binding to cholesterol in the membrane to form pore complexes; consequently, there has been limited research on its application to PDNVs. The Osmotic Shock Method and Hypotonic dialysis [170,191,192] are theoretically feasible for PDNVs, but lack supporting experimental data. Furthermore, saponins may cause cytotoxicity; therefore, their concentration and incubation time must be strictly controlled during use.

- Active loading methods

Electroporation

Electroporation is suitable for the loading of macromolecules (such as siRNA and miRNA), and its loading efficiency for nucleic acid-based drugs is generally higher than that of other methods [193]; however, the specific efficiency varies depending on the type of PDNVs and the optimisation of parameters (such as electric field strength, pulse duration and number of pulses). Inappropriate parameters may lead to drug aggregation or irreversible structural damage to the PDNVs, thereby reducing loading efficiency.

Regarding the loading of nucleic acid-based drugs, Rabienezhad et al. used electroporation (2000 V, 0.8 ms) to load siRNA into tangerine-derived nanovesicles for the treatment of colon cancer, achieving a loading efficiency of approximately 13% [193]; Yin et al. used electroporation (125 μF, 400 V, dual pulses) to load exogenous dsRNA into nanovesicles derived from Lane Late Navel oranges, achieving an efficiency of 6.0% [194]. The primary reason for this difference in efficiency may lie in the smaller size of siRNA molecules, whereas longer dsRNA strands are more readily able to enter the vesicles through the membrane pores formed by electroporation. For small-molecule chemical drugs, Mammadova et al. compared the efficacy of electroporation (750 V, 15 ms) and extrusion methods for loading tolvaptan into tomato-derived nanovesicles, finding electroporation to be significantly superior—when the mass ratio of nanovesicles (by protein mass) to tolvaptan was 1:1, the encapsulation efficiency reached 45%, with a drug loading of 37%, both of which were far higher than the 12% and 5% achieved by the extrusion method, respectively. Furthermore, the vesicle structure remained intact after electroporation, with a slight reduction in particle size but a uniform distribution, and stability was maintained after 6–12 months of storage at −80 °C. It is worth noting that the study explicitly stated that, regardless of whether tolvaptan was loaded via electroporation or extrusion, tomato-derived nanovesicles exhibited time-dependent and pH-dependent release [195]; however, it could not be confirmed whether PDNVs generally possess pH-dependent release characteristics.

Sonication-Assisted Loading

Sonication-assisted loading is best suited for loading hydrophobic small-molecule drugs into membrane-lipid-rich nanovesicles derived from fruits and vegetables, as the process is simple and rapid. However, it is not suitable for the direct loading of hydrophilic nucleic acids and macromolecular proteins; furthermore, high energy input may lead to the rupture, fusion, and loss of membrane proteins in the nanovesicles, thereby reducing particle yield and integrity [196]. In addition, during the process of increasing membrane permeability, sonication may non-specifically remove endogenous functional molecules (such as miRNAs and proteins) from the vesicles. Furthermore, loading efficiency is critically governed by several key parameters: plant source, drug properties, sonication time and power, drug-to-vesicle ratio, pulse mode, and ice-bath temperature control [178]; however, standardised protocols are currently lacking, posing challenges for batch-to-batch reproducibility.

This dependency is reflected in the markedly variable loading efficiencies reported across studies. For instance, Mammadova et al. compared the efficacy of three methods for loading curcumin into tomato-derived nanovesicles in an in vitro THP-1 cell model, finding that the direct incubation method yielded the highest encapsulation rate (0.22%), followed by the ultrasonic method (0.14%), and the extrusion method had the lowest (0.05%). It is worth noting that although the absolute loading efficiency of the sonication method remains relatively low, the drug-loaded vesicles prepared by this method outperformed those from the other two methods in inhibiting Lipopolysaccharide-induced IL-6 mRNA expression [197]. Zhou et al. employed sonication-assisted incubation to load sorafenib (SFB) into kiwifruit-derived nanovesicles, achieving an encapsulation efficiency of 69% and a drug loading of 6% by optimising the feed ratio (Knanovesicles:SFB = 10:1), and confirmed its liver-targeting antitumour effect and antitoxic effect in animal models [198]. 

Ultrasonic assistance is also used to construct vesicle-nanoparticle composites. Li et al. utilised sonication to load astaxanthin (AST)-loaded Poly(lactic-co-glycolic acid) cores (AST@PLGA) into broccoli-derived nanovesicles to construct AST@PLGA@BEVs. Under optimal conditions, The drug loading capacity of AST@PLGA@BEVs is 5.88%, and in vitro experiments demonstrated that the biological activity of AST was significantly enhanced due to various synergistic effects [199]. Mao et al. utilised sonication to facilitate the fusion of pre-loaded infliximab-carrying mesoporous silica nanoparticles with ginger-derived nanovesicles, thereby constructing a biomimetic nanocomposite with a core–shell structure. This enabled the colon-targeted delivery of the oral antibody, significantly improving the therapeutic efficacy in mice with colitis [200].

Freeze–Thaw Cycles

Freeze-thaw cycling is simple to operate, relatively mild in conditions, and holds potential for large-scale production. It is suitable for loading macromolecular drugs (e.g., enzymes, mRNA) as well as certain small-molecule drugs. Sharma et al. loaded ginkgetin (GGT) and berberine (BBR) into avocado-derived nanovesicles via three freeze–thaw cycles; fluorescence spectroscopy revealed a GGT loading efficiency of 64%, and 51.5% for BBR. The total loading efficiency (the arithmetic mean of the two drug loading efficiencies, as they were initially loaded in equal amounts) was 57.7% [201]. This method is straightforward and label-free, but it cannot distinguish between adsorption and true loading. PDNVs from different plant sources exhibit significant differences in sensitivity to freeze–thaw cycles; selecting plant sources with higher stability (such as olive and yam) is one effective strategy for improving storage stability [202]. However, even at −80 °C, repeated freeze–thaw cycles may still cause PDNV aggregation and fusion, membrane damage, RNA degradation, and lipid rearrangement [192,202,203]; the addition of appropriate cryoprotectant is an effective strategy to mitigate freeze–thaw damage [204]. Another drug delivery method utilising the principle of inducing membrane instability through temperature changes is thermal shock [192]; however, there are currently no reports of successful drug loading.

Extrusion

The core equipment for this method consists of a liposome extruder and polycarbonate membranes of varying pore sizes; drug loading is achieved by physically deforming the PDNV membrane through extrusion [195,197]. Extrusion effectively enhances the drug loading efficiency of nanovesicles and enables relatively precise control of their size distribution, making it suitable for loading proteins and nanoparticles [192], demonstrating application potential in fields such as vaccine delivery [192]. It should be noted that the mechanical forces generated by repeated extrusion may damage the functional proteins or lipid structures on the surface of PDNVs, thereby reducing their biological activity [205]. Therefore, when applying this method, it is recommended to balance drug loading efficiency against biological activity and optimise the number of extrusion cycles.

Mother Cell Modification Method

The mother cell modification method involves first introducing the target miRNA or siRNA into plant cells via Agrobacterium-mediated transformation or biolistic particle delivery. The transformed cells (or transgenic plants) subsequently secrete PDNVs containing the target molecules during culture, which are then isolated and purified from plant tissues or cell culture media [206,207]. Although this method is time-consuming and involves higher synthesis costs, it enables the enrichment of specific molecules at the source. Early research has largely focused on animal cells, and its application in plant cells is still in the exploratory stage; consequently, some researchers still prefer to use electroporation or transfection reagents to directly load RNA [208], but this strategy has demonstrated significant potential. In 2025, Emiliani et al. demonstrated, using a transgenic tobacco model stably expressing an exogenous gene, that the mRNA of the *Npt-II* gene was spontaneously packaged into PDNVs by plant cells without manual loading and was effectively protected therein, thereby providing direct and feasible evidence for the use of transgenic plants as oral siRNA bioreactors [206]. In the same year, Yury et al. genetically modified tobacco mother cells via *Agrobacterium*-mediated transformation to stably express an artificial miRNA targeting green fluorescent protein. Nanovesicles isolated from the transgenic leaves were naturally enriched with high levels of the targeted miRNA; these cargo-carrying nanovesicles were efficiently internalised by protoplasts and intact leaves, successfully inducing silencing of the target gene at both the transcriptional and protein levels [207].

Transfection Reagents

Transfection reagents (such as those based on cell-penetrating peptides) facilitate RNA loading into PDNVs by mediating interactions between nucleic acids and the vesicle membrane. In contrast, conventional liposome-based transfection methods rely on mechanisms that increase vesicle membrane permeability; however, their efficacy and specificity in the context of PDNVs remain unclear and may depend on the type of nucleic acid, the source of the PDNVs, and specific experimental conditions [208,209]. The ExoFect siRNA/miRNA Transfection Kit is specifically designed to load siRNA/miRNA into nanovesicles. It utilises a proprietary, improved cell-penetrating peptide technology to complex siRNA/miRNA and facilitate its entry into EVs, followed by the removal of free cell-penetrating peptides [208]. This method requires no electroporation equipment, is simple to perform, and leaves no residual free RNA after purification. To date, there have been very few reported cases of successful chemical transfection in the field of PDNVs. For example, Li et al. utilised the ExoFect Exosome Transfection Reagent to load siRNA targeting survivin into ginger-derived nanovesicles; the results indicated an siRNA encapsulation efficiency of 80%, and this delivery system effectively silenced the survivin gene in vitro; following intravenous injection in a tumour-bearing mouse model, it significantly inhibited tumour growth without toxicity [210].

Other potential drug delivery methods

Sonication and Extrusion-assisted Active Loading (SEAL) utilises ultrasonic treatment to facilitate the entry of ionisable molecules (such as ammonium sulphate) into extracellular vesicles, thereby establishing a transmembrane ion gradient; subsequently, the particles are homogenised through extrusion, and the irregular vesicle morphology is reshaped without compromising the integrity of the membrane structure. SEAL demonstrates universal and highly efficient loading capacity for ionisable hydrophilic molecules such as mitoxantrone and acridine orange; for instance, the encapsulation efficiency for DOX can exceed 60%, whereas values reported for other methods to date are mostly below 40% [178]. Notably, single-particle analysis via nano-flow cytometry (nFCM) indicates that only approximately 20% of particles actually achieve active loading. The authors attribute this discrepancy to the fact that the overall average encapsulation rate is primarily driven by a small number of highly loaded particles, whilst the majority of particles (approximately 82%) are underloaded or completely empty [178]. A comprehensive set of process parameters and analytical systems has been established for SEAL using milk-derived nanovesicles [178], providing a valuable reference for its extension to other sources of nanovesicles and offering the potential to overcome the bottleneck of low loading efficiency for hydrophilic drugs in PDNVs.

Takenaka et al. utilized an archaeal biotinylation reaction with a Gag-5BCCP and Cargo-BPL bifusion protein system to achieve multiply increased loading efficiency of peptides/proteins during the biogenesis of large extracellular vesicles (LEVs) [211]. This approach achieved a loading efficiency approximately five times higher than that of the conventional Gag fusion method [212]. However, this enhancement was observed only in LEVs; no significant improvement was detected in small extracellular vesicles (sEVs) [211]. This method holds promise for overcoming the loading bottleneck of PDNVs for protein-based drugs; however, PDNVs lack a defined inner membrane anchoring protein, and the establishment of genetic transformation systems from different plant sources is time-consuming and complex, potentially undermining the core advantages of PDNVs—namely, their natural origin and low cost. Furthermore, the gastrointestinal stability of fusion proteins in oral delivery scenarios and the impact of exogenous protein modifications on the natural targeting properties of PDNVs remain unverified. Consequently, this method is better suited as a conceptual reference for long-term basic research rather than a technical solution that can be readily implemented in the short term.

The Cubosome-mediated membrane fusion method utilises cubosomes with a positive Gaussian modulus, which fuse spontaneously upon contact with ordinary lipid membranes due to the release of intrinsic stress. Drug loading is achieved through simple mixing in deionized water for just 10 min, realising a loading efficiency of nearly 100% for macromolecules such as mRNA and IgG, whilst the fused hybrid exosomes retain the targeting properties of the source cells [213]. The method’s membrane-source-independent nature and mild aqueous operating conditions are highly compatible with the fragile membrane structure of PDNVs, potentially offering a novel approach for loading macromolecular drugs into PDNVs. However, the glycolipids abundant in PDNV membranes, such as MGDG and DGDG, may affect fusion efficiency. In the context of oral delivery, both the stability of the Cubosome’s lipid components in the gastrointestinal tract and whether the natural targeting properties of PDNVs are retained following fusion require systematic validation.

Zhang et al. subjected freeze-dried *Spirulina platensis* (SP) microalgae to a rehydration process, utilising the osmotic pressure difference during freeze-drying and rehydration to efficiently incorporate amifostine into the cells. The experiments demonstrated that the loading efficiency (approximately 65%) was significantly higher than that of fresh SP (approximately 30%), and under optimal conditions, it could reach more than twice that of fresh SP [214]. This drug-loading strategy preserves the natural spiral structure and dense cell wall of SP, enabling it to withstand gastric acid erosion and achieve uniform drug distribution throughout the small intestine [214]. This suggests that although the membrane structure of PDNV is more fragile and challenges such as ice crystal damage need to be addressed, the integrated freeze-drying preservation and drug loading of PDNVs is potentially feasible through the optimisation of freeze-drying protectants and rehydration conditions.

Hillman et al. proposed loading the CRISPR/Cas9 system itself (in the form of plasmids, mRNA or RNPs) into PDNVs, utilising the natural targeting and barrier-crossing capabilities of PDNVs to achieve gene editing therapy in specific tissues or cells in vivo [215]. However, this approach remains at the theoretical discussion stage and requires further experimental validation.

If the focus is on functional validation and mechanistic investigation, the use of a kit is recommended. This method offers high encapsulation efficiency, effective gene silencing, clear in vivo antitumour activity and good reproducibility, making it suitable for rapid progression from in vitro screening to in vivo validation [210,216]. If the focus is more on clinical translation, a trade-off must be considered: the incubation method is highly safe and simple to operate, and is suitable for hydrophobic small molecules, but its efficiency is limited; efficiency can be improved through pH optimisation. Electroporation is suitable for nucleic acid loading, but may damage vesicle structures and requires optimisation of the buffer to reduce interference caused by precipitation. For proteins and nanoparticles, high-efficiency methods such as Sonication-Assisted Loading and Extrusion can be selected, but these may alter the activity of membrane proteins [192]. It is worth noting that due to compositional differences, PDNVs from different sources exhibit varying loading efficiencies when using the same loading strategy [190]; therefore, different loading methods must be adapted [193]. Furthermore, for PDNVs prepared via passive incubation or sonication-assisted loading, gel filtration [178,217] or ultracentrifugation [198] is commonly employed to remove unencapsulated free drug.

In PDNV drug-loading methods, the issue of false positives in loading efficiency is almost universally present. This problem stems primarily from two sources. Firstly, drug aggregation can lead to an overestimation of efficiency. For example, saponins themselves form micelles that encapsulate hydrophobic drugs, creating a false impression of loading efficiency. Studies have shown that ultrafiltration does not completely remove drug micelles, with residual levels reaching up to 9.4 μM, whereas SEC can reduce free drug residues to 0.09 μM; consequently, SEC is regarded as the current gold standard for eliminating false-positive signals from free drugs [218]. Another study proposed the sponge cell method, which efficiently adsorbs free drug by co-incubating drug-loaded small extracellular vesicles with donor cells for 15 min [219]. This method offers the advantages of being rapid, cost-free and simple to operate; however, it lacks versatility (as it requires the use of donor cells) and may carry a risk of cross-contamination. Consequently, it is more suitable as a preliminary alternative to traditional purification methods (such as SEC) rather than as a standard protocol. Furthermore, it is difficult to distinguish between surface adsorption and internal loading. Drugs may be adsorbed onto the surface of EVs rather than truly entering the lumen, thereby altering their release behaviour and in vivo fate. To diagnose aggregation issues, validation at the single-particle level and the use of critical controls can be implemented [220,221]. Optimising the drug-loading process at the source is a viable solution. To address the issue of siRNA aggregation commonly associated with electroporation, gentler passive diffusion methods may be selected, or the buffer system may be optimised (e.g., by adding EDTA) [222]; furthermore, for large-scale processing, SEC or tangential flow filtration may be employed, with the combination of both yielding the best results [70].

#### 4.1.2. Engineering Modification Technology

**Figure 7:**
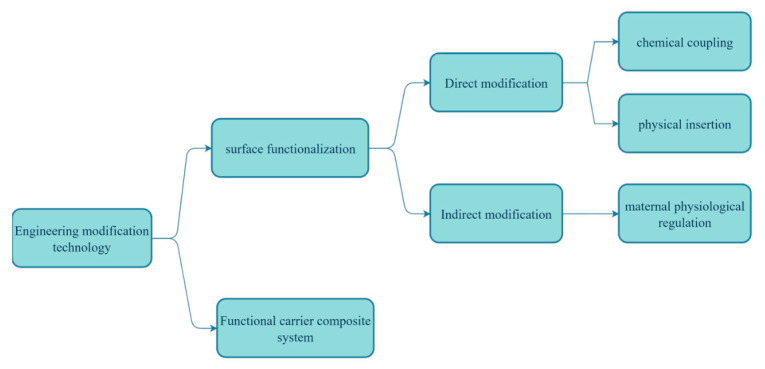
Engineering modification technology.

Surface modification focuses on the functionalisation of PDNV surfaces, such as the conjugation of targeting ligands or antibodies; this can be a natural consequence of lipid reorganisation, such as charge regulation, or serve as a means of functional expansion following lipid reorganisation. Lipid engineering, meanwhile, serves as the underlying technical framework for modification. By adjusting the lipid composition—such as cholesterol, charged lipids or integrated membrane proteins—it fundamentally alters the structure and surface properties of the vesicles, including their charge and stability. The two approaches work in concert to optimise PDNVs performance. Techniques such as charge regulation, chemical conjugation, membrane fusion and hybridisation serve as specific tools for achieving lipid engineering and surface modification (Figure 7).

##### Surface Functionalisation

Surface functionalisation of PDNVs is a core technology that modifies their membrane structure through genetic engineering or chemical and physical means to enhance targeting, stability and biological activity. Depending on the nature of the modification target, these methods can be broadly categorised into direct and indirect modification. Both approaches achieve functional optimisation by regulating membrane proteins, lipids or ligand molecules, and demonstrate significant potential in disease treatment and drug delivery.

- Direct modification

Direct modification involves surface modification of the isolated and purified nanovesicles themselves through chemical, physical or biological means, without relying on the modification of the parent cells.

Chemical Coupling

Chemical coupling is one of the most commonly used strategies for direct modification. It involves attaching functional molecules to the surface of PDNVs via covalent or non-covalent interactions, thereby controlling the loading quantity and binding sites of functionalised molecules on PDNVs to achieve functional modifications such as targeted delivery and drug tracing [105]. Covalent and non-covalent modifications represent the two primary approaches, with significant differences between them in terms of bond stability, reaction mildness, and the types of ligands that can be employed. Covalent modification utilises active groups on the PDNV surface, such as amino and carboxyl groups, to form stable chemical bonds via click chemistry (e.g., copper-catalyzed azide-alkyne cycloaddition) or chemical crosslinkers (e.g., 1-Ethyl-3-(3-dimethylaminopropyl)carbodiimide/N-Hydroxysuccinimide[EDC/NHS]), enabling the efficient attachment of ligands such as antibodies and adaptors [48,76,105,223]; however, this may compromise vesicle integrity [224]. Non-covalent modification utilises physical interactions such as electrostatic adsorption, hydrophobic insertion or ligand-receptor specific recognition to achieve reversible anchoring of functional molecules under mild conditions [48,76,105,223]. This approach is suitable for peptide molecules but exhibits lower in vivo stability.

Regarding covalent modification, Tang et al. covalently conjugated human epidermal growth factor receptor 2(HER2) to grapefruit-derived nanovesicles; in vitro experiments confirmed that this promotes the internalisation and accumulation of grapefruit-derived nanovesicles in HER2-positive breast cancer cells and helps to overcome trastuzumab resistance [225]. Unlike Tang et al., who conducted only in vitro experiments, Long et al. covalently attached pre-synthesised cyclic Arginine-Glycine-Aspartic acid(cRGD)-targeted doxorubicin nanoparticles (DN) to the surface of orange-derived nanovesicles via an EDC/NHS-mediated amidation reaction, thereby constructing the targeted nanomedicine DN@OEV. DN@OEV enhances cellular uptake via cRGD targeting and hijacks the endocytic pathway to achieve highly efficient endocytotic transport. In both 3D tumour spheroids and mouse in situ ovarian cancer models, it demonstrated superior penetration, tumour accumulation and antitumour efficacy compared to free drugs and nanoparticles, and this was dependent on active transport rather than the passive enhanced permeability and retention (EPR) effect [226].

In terms of non-covalent modification, Tan et al. anchored the REDV peptide(Arg-Glu-Asp-Val) to ginseng-derived nanovesicles to construct targeted nanovesicles (RGE), and embedded these into a hyaluronic acid and polyether F127 (Pluronic^®^ F-127) co-crosslinked thermoelectric hydrogel (HFN) to form HFN-RGE; This system utilises the directed biomimetic electric field generated by HFN to guide the migration of epithelial and fibroblast cells, whilst simultaneously controlling the release of RGE to specifically reverse endothelial dysfunction, thereby synergistically promoting the repair of diabetic ulcers [227].

It is important to note that peptide conjugation refers to the process of attaching functional peptides (such as targeting peptides or transmembrane peptides) to the surface of nanovesicles via covalent or non-covalent interactions, using chemical or enzymatic methods, to achieve specific functions [228,229,230]; whether this constitutes covalent or non-covalent modification depends on the specific linking strategy employed. Due to its cyclic structure, which locks integrin binding into the optimal conformation, cyclic Arginine-Glycine-Aspartic acid (cRGD) exhibits approximately 1000-fold higher affinity for αvβ3 integrin than linear (RGD), as well as superior protease resistance; consequently, it is the preferred choice in the field of tumour targeting and is now gradually being applied to PDNVs [226], whereas the linear RGD peptide is significantly limited in its application due to poor stability and insufficient affinity [231,232]. Compared to conventional targeting molecules, the core advantage of cRGD lies not only in its ability to mediate targeted recognition, but also in its capacity to hijack the endosomal recycling pathway of integrins, thereby transforming ordinary carriers into an active transport system capable of progressive, deep tissue penetration. This offers unique value in overcoming the delivery barriers of solid tumours [226,233].

Physical insertion

Physical insertion is primarily categorised into lipid anchoring, membrane fusion and hybridisation [76].

Hydrophobic insertion is a form of lipid anchoring technology. It utilises the hydrophobicity of the PDNV lipid bilayer to insert ligands bearing hydrophobic groups into the bilayer, whilst simultaneously directing various functional ligands to the PDNV surface to achieve functional modification. This method is relatively simple to perform and does not significantly affect the activity of the ligands. Han et al. constructed the FA-GDEVs system by inserting FA-PEG2000-Chol (folic acid-polyethylene glycol 2000-cholesterol) into the lipid bilayer membrane of ginger-derived nanovesicles. FA-GDEVs achieve targeted delivery by utilising surface-modified folic acid to specifically bind to folate receptors (FRs), which are highly expressed on M1 macrophages in inflamed joints. In a collagen-induced arthritis mouse model, treatment with FA-GDEVs significantly reduced arthritis clinical scores, minimised cartilage damage and bone erosion, and inhibited osteoclast activity [234]. Furthermore, PEGylation significantly prolongs the half-life of PDNVs in the bloodstream, reduces hepatic clearance, and enhances accumulation in target organs. Zhang et al. isolated nanovesicles from Asparagus cochinchinensis (ACNVs); to further optimise their in vivo performance, they conjugated 2 mg (6.7 wt.%) DSPE-PEG2000 (1,2-distearoyl-sn-glycero-3-phosphoethanolamine-N-[methoxy(polyethylene glycol)-2000]) into the ACNV lipid membrane to prepare PEG-ACNVs. In a tumour-bearing mouse model, intravenous injection of PEG-ACNVs not only significantly prolonged the circulation time and enhanced tumour-targeted accumulation, but also effectively inhibited tumour growth, with no significant systemic toxic side effects observed [235].

Membrane fusion and hybridization technology is a drug delivery method that primarily involves fusion with artificial liposomes and hybridization with cell membranes, offering significant advantages in the delivery of hydrophobic drugs. This technology effectively combines the excellent drug encapsulation capacity of liposomes with the barrier-crossing delivery capability of PDNVs; the composite carriers formed by this combination can better encapsulate hydrophobic drugs and deliver them efficiently to target tissues. Its application in PDNV engineering has become widespread, and the membrane hybridisation methods currently successfully employed for PDNVs primarily include mixed induction fusion and ultrasonic fusion. For example, Anti-inflammatory *Hydrangea macrophylla* leaf-derived nanovesicles (HML-EVs) were fused with liposomes loaded with terpinen-4-ol/azelamide monoethanolamine via a mild mixing-induced fusion method, and successfully constructed into an HML-EV/liposome hybrid system by Ryu et al. In a UV radiation-induced reconstructed human skin (RHS) inflammation model, treatment with this hybrid system resulted in a significant reduction in the expression levels of key inflammatory mediators compared to the UV-stimulated control group, as revealed by immunofluorescence staining [236]; Yang et al. utilised ultrasonic fusion to embed 4T1 breast cancer cell membrane fragments into lemon-derived nanovesicles and loaded them with DOX, thereby constructing Lemon-derived Extracellular Vesicles fused with 4T1 cell membrane fragments loaded with Doxorubicin (LEVBD). LEVBD retained tumour membrane marker proteins such as CD44 and CD47, had a plasma half-life of 3 h, and exhibited a 5.7-fold increase in targeted uptake of homologous tumours; in a mouse dual-tumour model, it specifically homing to 4T1 tumours and significantly inhibited tumour growth [237]. Compared to ultrasonic fusion, mild mixed induction fusion offers milder conditions and simpler operation, whilst better preserving the natural activity of PDNVs; it is suitable for scenarios requiring high vesicle integrity, but lacks high-throughput capability. There are differences in validation strategies between the two; (FRET) assays and colocalisation imaging are both mainstream techniques for verifying successful membrane fusion [238]. Quantitative FRET used by Ryu et al. [236] can accurately quantify fusion efficiency, whereas semi-quantitative co-localization can only confirm whether fusion is successful, making it difficult to compare the fusion efficiencies of the two methods. Theoretically, the freeze–thaw method could also be applied to PDNVs [239]; however, due to their high content of unsaturated fatty acids and low tolerance to low temperatures, the addition of protective agents and optimisation of freeze–thaw parameters are required for their application.

A combination strategy of chemical conjugation and physical insertion

Chemical conjugation is often combined with physical insertion to achieve complementary functionality. The distearoylphosphoethanolamine (DSPE) functionalisation platform (including DSPE and DMPE) is a typical example. Its hydrophobic tail (DSPE) can stably embed itself into the lipid bilayer membrane of PDNVs, whilst terminal functional groups (such as Rabies virus glycoprotein(RVG) or maleimide) covalently conjugate to target molecules. Furthermore, the steric hindrance and charge-masking effects of the PEG chains prolong circulation time in vivo and reduce immune clearance, thereby enabling the efficient targeted delivery and stable engineering of PDNVs [240]. Xu et al. isolated Pueraria lobata-derived nanovesicles (Pu-Exos) and anchored the DSPE-PEG-RVG (DPR) ternary ligand onto their surface via hydrophobic insertion, thereby constructing a brain-targeted engineered PDNV designated as Pu-Exos-PR. In vivo distribution experiments indicated that, 6 h after intravenous injection, the accumulation of Pu-Exos-PR in the mouse brain was approximately 1.45-fold higher than that in the unmodified group. They hypothesised that the mechanism involved RVG-mediated targeting of nicotinic acetylcholine receptors and PEG-induced prolongation of circulation time; however, these mechanisms have not yet been directly experimentally validated [23]. DSPE-PEG2000-Maleimide was covalently linked to the thiol groups on the aptamer via a maleimide-thiol click reaction, thereby attaching the aptamer to the PDNV surface and significantly enhancing its targeting ability. Moon et al. incorporated DSPE-PEG2000-Maleimide into the membrane of grapefruit-derived nanovesicles via hydrophobic insertion; subsequently, the maleimide group was conjugated with the 3′-thiol-modified R11-3 aptamer via click chemistry, successfully constructing the targeted pEV-R11-3. In vitro cell uptake experiments demonstrated that, in the human brain endothelial cell line hCMEC/D3, the uptake of pEV-R11-3 was approximately twofold higher than that of unmodified PDNVs, and its affinity for endothelial cells was higher than that for the glioblastoma U87MG cell line, suggesting its potential for targeting blood–brain barrier endothelial cells [240]. 1,2-Dimyristoyl-sn-glycero-3-phosphoethanolamine (DMPE) has been demonstrated to be suitable for the surface engineering of M-EVs and immune cells; however, its direct application in PDNVs has not yet been reported. From a mechanistic perspective, the application of DMPE for the engineering modification of PDNVs also appears feasible. In a study by Kim et al. [241], using the human MDA-MB-231 triple-negative breast cancer cell line as a model under in vitro cell culture conditions, initial coating efficiency and membrane retention time were evaluated as key parameters. DMPE was found to outperform both DSPE and cholesterol in these metrics, leading to its identification as the optimal lipid anchor in that system [241]. It should be noted that this study demonstrated that DMPE performs better at the engineering application level, i.e., it anchors a greater number of molecules per unit time and remains on the membrane for a longer duration; however, the thermodynamic affinity constant was not directly measured. From the perspective of lipid chemistry, DSPE has a longer hydrophobic chain (C18), and its monomolecular anchoring stability should, in theory, be higher; however, the superior performance of DMPE under these experimental conditions may stem from its shorter hydrophobic chains (C14), which produce a smaller conical volume and potentially increase its propensity for membrane insertion in triple-negative breast cancer cell [241]. The true difference in thermodynamic affinity between the two still requires direct verification.

PDNVs can also adopt surface charge modification strategies used for nanoliposomes; this not only addresses the low loading efficiency of native PDNVs but also enhances cellular uptake and endosomal escape, providing a practical and feasible optimisation pathway for the application of PDNVs as nucleic acid drug delivery carriers [242].

- Indirect modification

Indirect modification does not directly alter the membrane structure or chemical composition of PDNVs, but rather indirectly influences surface charge, targeting, or the distribution of functional molecules by regulating their biogenic environment, the state of the parent cell, or the action of external carriers. Indirect modification methods for PDNVs surface functionalisation primarily include genetic engineering regulation and cell culture medium regulation.

Maternal physiological regulation

The pH of the plant cell culture environment can influence the physicochemical properties of the secreted PDNVs. Studies have shown that culture medium pH can influence the loading efficiency of secondary metabolites into EVs by regulating their ionisation state. Taking acidic conditions at pH 5.8 as an example, the proportion of weakly acidic molecules (such as caffeic acid derivatives) existing in a neutral form increases, making them more likely to enter vesicles via passive diffusion; conversely, alkaline conditions cause molecules to become charged, hindering membrane penetration [162]. This phenomenon is consistent with the principle that pH regulates the ionisation state of metabolites and their membrane permeability. Furthermore, changes in intracellular pH have been shown to correlate with the production of secondary metabolites, suggesting that culture medium pH may indirectly regulate the composition of EVs by influencing intracellular pH homeostasis [162].

##### Functional Carrier Composite System

Combined therapy using PDNVs and medical devices such as hydrogels has also emerged as a current trend. This approach utilises hydrogel carriers to address the issues of PDNVs being easily cleared and struggling to retain at the target site, thereby achieving local anchoring and sustained release of PDNVs. It simultaneously integrates the mechanical support provided by hydrogels with the biological activity of PDNVs, synergistically enhancing therapeutic efficacy through both physical fixation and biological regulation. This is particularly suitable for the repair needs of complex pathological microenvironments, such as diabetic fractures and acute full-thickness skin defects [243,244,245,246,247].

Compared to the physical anchoring of hydrogels, Metal-organic frameworks (MOFs)-based composite carriers offer a designable stimulus-responsive delivery strategy. MOFs are crystalline porous materials formed by the coordination of metal ions or clusters with organic ligands, characterised by high specific surface area, tunable pore sizes, abundant active sites and dynamic responsiveness. Kong et al. combined Cu-MOF nanodots with cucumber-derived nanovesicles and, following RGD modification, constructed the NDs@EV-RGD platform. This platform utilises the high deformability of cucumber-derived nanovesicles to penetrate the stratum corneum; via RGD, it targets and accumulates in hypertrophic scars. Upon near-infrared light triggering, it generates reactive oxygen species to induce fibroblast apoptosis and deplete glutathione to enhance photodynamic efficacy, significantly promoting collagen remodelling in a hypertrophic scar model in rabbit ears [231]. From a design perspective, MOFs provide high drug-loading capacity and programmable release (e.g., ligand protonation triggered by the slightly acidic pH of tumours, or destruction of the MOF structure by near-infrared photothermal effects [248,249]), whilst PDNVs contribute targeting, low toxicity and tissue permeability; the two are complementary, and there have already been explorations into enhancing the therapeutic potential for central nervous system diseases [250,251]. However, MOF-based delivery systems currently face the limitation of high costs. Furthermore, safety and stability are difficult to guarantee. The potential for metal leakage from MOFs, combined with the plant-derived proteins in PDNVs, may induce synergistic toxicity or unexpected immune responses, and long-term safety remains unclear [252]; simultaneously, MOFs are prone to premature degradation in physiological environments [253], whilst the membrane structure of PDNVs is fragile. Following their combination, there is a lack of effective regulation regarding interfacial bonding strength and synchronised in vivo release behaviour [254], making it difficult to achieve the anticipated synergistic controlled-release effect. Finally, large-scale production represents a common bottleneck. PDNVs themselves suffer from significant batch-to-batch variability and low yields; the integration with carriers such as MOFs involves complex multi-step processes, further exacerbating the challenges of standardised production and clinical translation.

In the context of oral delivery, PDNVs also demonstrate potential as functional carrier composite systems. Cathrine’s team discovered that fenugreek-derived nanovesicles (FGDNVs) contain plant ferritin with a natural nanocage structure capable of improving iron-deficiency anaemia. More importantly, it resists protease K degradation and mimics gastrointestinal digestion (gastric acid, digestive enzymes), exhibiting significantly greater stability than free pea ferritin (PF) [255], whilst retaining the biological advantages of PDNVs; consequently, FGDNVs may be considered a superior carrier for oral nanomedicine delivery systems. Theoretically,genetic or chemical engineering modifications could further enhance the targeting affinity of ferritin. However, the drug-carrying effects and carrier compatibility remain unclear. Whilst FGDNVs possess therapeutic efficacy in improving anaemia, there is a lack of validation regarding whether the two components exhibit synergistic effects or interfere with one another when other drugs are loaded; In addition, drug loading may alter ferritin conformation, affecting its iron release kinetics or resistance to gastrointestinal digestion. Although FGDNVs possess a natural nanocage structure, systematic studies on the binding mechanism between plant ferritin and exogenous drugs, as well as encapsulation efficiency and stability, remain lacking. With a similar objective, Xiao and Wang et al. developed an oral delivery system (PEG@SN-MNs), which achieves multi-mechanism synergistic treatment of N-Acetyl-para-aminophenol-induced liver injury by integrating the gastrointestinal stability and liver-targeting properties of mulberry leaf-derived exosome-like nanoparticles (MNs), the immunoprotective effects of PEG modification, and the pharmacological activity of Silymarin nanocrystalss(SN) [256].

The core issue currently facing the field of engineered modification of PDNVs is not a lack of technical methods, but rather a lack of critical scrutiny of their actual efficacy and exploration of optimisation strategies. Most studies merely report successful modification without systematically evaluating the impact of such modifications on PDNV membrane integrity and biological activity. Direct modification is currently the mainstream strategy for surface functionalisation of PDNVs. However, while covalent modification enables efficient ligand attachment, it may compromise vesicle integrity [224]; conversely, non-covalent modification offers mild conditions but suffers from limited in vivo stability. Among physical embedding techniques, membrane fusion methods using mild induction better preserve the natural activity of PDNVs but lack high-throughput capability, whilst ultrasonic methods are more efficient but may disrupt membrane protein function [236,237,238]; to date, there has been no direct comparison of the two under standardised conditions. The difference in anchoring efficiency between DMPE and DSPE in PDNVs remains to be further verified [241]. Indirect modification influences the physicochemical properties and content composition of PDNVs via culture medium pH [162]. However, this strategy currently remains at the level of phenomenological description, and its mechanism has yet to be elucidated. Compared with direct modification, indirect modification suffers from poor controllability and an unclear mechanism; it cannot yet serve as an independent engineering approach, but rather functions primarily as a supplement to direct modification.

In summary, the field urgently needs to shift from ‘demonstrating feasibility’ to ‘quantitative evaluation’—including a systematic trade-off between modification efficiency and side effects, differences in the response of PDNVs from different sources to the same modification strategy, and a functional comparison between modified vesicles and native vesicles. Otherwise, a large number of studies will remain a mere accumulation of feasibility cases, making it difficult to support genuine clinical translation.

#### 4.1.3. Marking Method

Fluorescent dye labelling is the most fundamental and commonly used method in studies of PDNVs cellular uptake [257,258,259]. Lipophilic dyes such as PKH67 achieve labelling by embedding themselves within the membrane lipid bilayer, and are used to track the cellular uptake of PDNVs [257]. However, their limitation lies in the inability to distinguish between intact nanovesicles and fragments, and free dyes are prone to forming micelles, leading to false positives—a problem that is common among lipophilic dyes, yet one that has not been given sufficient attention in the methodological validation of most PDNVs studies. Calcein AM, acting as a content probe, is non-fluorescent in its native state; upon entering intact nanovesicles, it is hydrolysed by internal esterases into green-fluorescent calcein and retained within the lumen, thereby labelling only intact, functional PDNVs [260], Its advantage lies in the ability to exclude interference from ruptured vesicles and debris; however, free dye must be rigorously removed, and it cannot detect subsequent changes in the integrity of labelled vesicles. Potomac Gold is also a lipophilic dye, with a mechanism similar to that of PKH67 [257]. Steć et al. [180] conducted a comparative study on nanovesicles derived from Citrus limon, which further revealed the differences between various labelling strategies: SYBR Gold (a nucleic acid dye) produces a strong signal, requires no purification, and does not alter the particle size or zeta potential of the vesicles; it is therefore recommended as the dye of choice for capillary electrophoresis analysis of PDNVs. In contrast, carboxyfluorescein diacetate succinimidyl ester and CellMask (a lipid dye), whilst capable of labelling, both alter the physicochemical properties of the vesicles [180]. It is worth noting that this study was based on a capillary electrophoresis platform, and the transferability of its conclusions to conventional cell uptake experiments has not yet been verified. DiI, DiD, DiO and DiR have been widely used in M-EVs, but there are no reports of their direct application in the field of PDNVs [261,262,263,264,265,266]—this represents both a methodological gap and suggests that differences in the membrane composition of PDNVs (such as high levels of glycolipids) compared to M-EVs may result in varying dye suitability.

In contrast to the traditional fluorescent dye strategies described above, the ultra-small (1.8 nm) multifunctional quantum dots (Ag_2_Se@Mn QDs) developed by Zhao et al. are loaded directly into the lumen of nanovesicles via electroporation. This approach eliminates the need for donor cell pre-labelling, achieves universal labelling with >90% efficiency within minutes, and provides dual-modality imaging capabilities (near-infrared fluorescence and magnetic resonance imaging, MRI) from a single particle, with minimal impact on the intrinsic functions of the vesicles [267]. However, as this method has been validated using mammalian microvesicles (MVs), its applicability to PDNVs requires further validation.

## 5. Disease Transformation and Application

**Figure 8:**
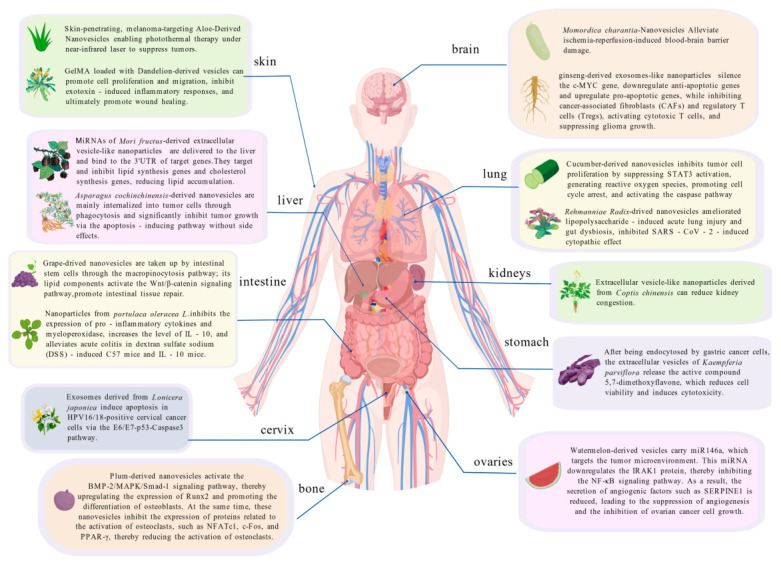
Some PDNVs and their functions. This picture is adapted from Figure 4 of reference [169].

This review compiled a table containing nearly 120 entries covering PDNV sources, isolation methods, morphology, particle size, zeta potential, functions and mechanisms, active components, and sites of action. Due to the extensive content, the complete table is not presented in the main text and only partial information is shown (Figure 8, Supplemental Tables S2 and S3).

### 5.1. Administration Route Selection

Extensive research has demonstrated that PDNVs can be taken up by mammalian cells via phagocytosis and endocytosis; their lipid composition regulates the efficiency of cellular uptake, exhibiting broad adaptability under both normal and pathological conditions [268,269,270]. Elucidating the relevant mechanisms is of great significance for optimising the biotherapeutic and drug delivery applications of PDNVs. The selection of the route of administration is a key consideration in the clinical translation of PDNVs; its appropriateness directly influences the in vivo distribution, bioavailability, targeted delivery efficiency and in vivo safety of the nanovesicles, thereby determining the ultimate clinical value of the therapeutic regimen. There are significant differences in the in vivo fate and application scenarios of different routes. In inflammatory regions, where proteins such as transferrin and eosinophil cationic protein are abundant, negatively charged PDNVs can preferentially adhere to inflammatory sites via electrostatic interactions, exhibiting a degree of passive targeting [271,272].

#### 5.1.1. Topical Administration

PDNVs can achieve transdermal administration via pathways such as hair follicles, sebaceous ducts and associated appendages (sebaceous/sweat glands) [273], possess the ability to penetrate the stratum corneum [274], and are suitable for skin defect repair and the transdermal delivery of active ingredients [275]. Loading PDNVs into hydrogels can improve their storage stability, prolong their retention time on the skin and enable sustained release, thereby enhancing transdermal delivery efficiency [153]. Cucumber-derived nanovesicles isolated by Abraham et al. [276] demonstrated approximately a two-fold increase in both the transdermal penetration rate and penetration depth of the lipophilic model drug they carried, compared to the free drug control group in an ex vivo porcine skin model, achieving effective delivery to the active dermis [231]. It should be noted that although PDNVs prepared by high-pressure homogenisation (HPH) can increase penetration by 1.5-fold [231], the HPH products contain a large amount of plant debris, making them difficult to purify using conventional methods; their presence must be indirectly confirmed through functional experiments. However, there are few studies systematically comparing the transdermal efficiency of different strategies. Furthermore, although physical methods such as microneedles can be employed to enhance transdermal penetration [277], the specific mechanism by which PDNVs cross the skin barrier requires further investigation.

Lemon-derived nanovesicles, when administered via intratumoural injection, can inhibit the growth of mouse chronic myeloid leukaemia xenografts, and intratumoural injection exhibits stronger activation of the TNF-related apoptosis-inducing ligand (TRAIL) apoptosis pathway [278]. Yang et al. fabricated injectable hydrogels from ginseng- and spinach-derived nanovesicles [279]. Upon intratumoral injection into murine tumor models, calcium-triggered in situ gelation enabled sustained local release, achieving ~80% tumor inhibition, suppressing lung metastasis, and activating long-term immunological memory. These two approaches represent distinct paradigms within the intratumoural delivery strategy for PDNVs. The former focuses on the direct application of natural vesicles, with antitumour effects primarily dependent on the vesicles’ inherent biological activity and a relatively simple mechanism of action; the latter, through functional carrier composite strategy, significantly enhances therapeutic efficacy and successfully induces systemic antitumour immunity and long-term immune memory, surpassing the direct application of natural vesicles in both depth and persistence of action.

The most notable advantage of scaffolds is their ability to facilitate the local sustained release of drugs. In a study by Wang et al., goji-derived nanovesicles loaded with isoliquiritigenin were encapsulated within a 3D-printed gelatin methacryloyl scaffold; in vitro, they sustained drug release for up to 16 days and exhibited significant controlled-release properties under various pH conditions. Following implantation of this composite scaffold into a rat model of spinal cord injury, it effectively modulated the post-injury inflammatory response and promoted the repair of damaged axons and the recovery of neural function [280]. Unlike drugs administered orally or intravenously, which are rapidly distributed and metabolised systemically, drugs released from the implanted scaffold are released slowly and locally at the site of injury, maintaining effective concentrations for several days to weeks. This enhances local therapeutic efficacy whilst reducing the risk of systemic side effects. However, the scaffold involves a multi-step process including 3D printing, encapsulation and sterilisation, presenting challenges regarding batch consistency and large-scale production; furthermore, the long-term biosafety of the implant material requires systematic evaluation. It should be noted that this strategy is only applicable to localised lesions with a clearly defined location that can be surgically exposed, thus having a relatively narrow scope of application. Additionally, this study only followed up to 8 weeks post-surgery and did not assess long-term efficacy and safety. Consequently, scaffold-based delivery should currently be positioned as a supplementary strategy for specific indications, rather than a mainstream route of administration.

Intranasal instillation is one of the key methods for the respiratory administration of PDNVs, primarily comprising intranasal instillation. Intranasal instillation can bypass the blood–brain barrier via the olfactory and trigeminal nerve pathways to achieve targeted delivery to brain lesions, making it suitable for the treatment of neurological disorders [281]. Umezawa et al. loaded exogenous microRNA (cel-miR-39) into PDNVs isolated from onions, cherry tomatoes, grapes and grapefruit, and administered them to adult male C57BL/6J mice via intranasal instillation and intravenous injection, respectively, assessing microRNA delivery efficiency by quantitative RT-PCR [282]. The results indicated that intranasal instillation was more effective than intravenous injection in delivering microRNA to the brain, and that onion-derived nanovesicles were the most effective carriers for delivery to the olfactory bulb and caudal brain regions [282]. Dad et al. further confirmed that following intranasal administration, the fluorescent signal from grapefruit-derived nanovesicles was primarily distributed in the lungs and brain, suggesting their potential for targeting the central nervous system via the trans-nasal-brain pathway [119,283]. However, the application of intranasal instillation still faces several challenges. First, mucociliary clearance can remove drugs from the nasal cavity within a short period of time, resulting in an extremely short retention time at the absorption site [284]. Second, the single administration volume is generally limited to less than 200 μL per nostril; exceeding this volume may cause the drug to flow out of the nostril or into the throat, making high-dose delivery difficult [285]; furthermore, the nasal mucosa contains abundant metabolic enzymes (including cytochrome P450 enzymes, peptidases, esterases, etc.), which theoretically could degrade the surface proteins or lipid bilayer of PDNVs. However, the phospholipid bilayer structure of PDNVs has been shown to protect the internal contents from degradation by gastrointestinal digestive enzymes during oral administration [286]. Based on the same principle of physical isolation, PDNVs may also possess a certain degree of resistance to nasal mucosal enzymes. However, there are currently very few studies directly evaluating the impact of nasal mucosal metabolic enzymes on the stability of PDNVs.

Within the broader field of nanodelivery systems, significant progress has been made in nebulisation inhalation techniques. Due to their low shear force, non-thermal nature and controllable particle size, vibrating mesh nebulisers are widely used for the pulmonary delivery of synthetic carriers such as liposomes and polymeric nanoparticles; the successful application of the COVID-19 nebulised inhalation vaccine (Ad5-nCoV) further validates the clinical feasibility of this approach [287]. Compared with synthetic liposomes, the natural membrane structure of PDNVs may offer superior biocompatibility. However, research on the nebulised inhalation of PDNVs remains largely unexplored: whether the unique membrane composition of PDNVs (rich in phytosterols and lacking cholesterol) affects their nebulisation stability, the impact of the nebulisation process on vesicle integrity, and the relationship between formulation parameters (such as particle size, viscosity and surface charge) and nebulisation efficiency all require systematic investigation.

PDNVs remain in the early exploratory stages in the field of ocular administration. Research on synthetic carriers such as liposomes indicates that nanoscale dimensions, positively charged surface modification, and sustained-release properties are key design elements for ocular delivery systems [288]. The corneal penetration efficiency of nanoparticles is influenced by the integrity of the corneal barrier. Nanoparticles of approximately 200 nm can effectively traverse the tight junction-barrier of the corneal epithelium, enabling intraocular delivery [289]. In cases where the corneal barrier is compromised (e.g., by alkali burns), even nanoparticles larger than 600 nm can penetrate the corneal tissue and exert therapeutic effects [290]. Bao et al. isolated nanoparticles from the protocorm-like bodies of *Dendrobium officinale*; in vitro experiments demonstrated that these nanoparticles can protect human corneal epithelial cells from hyperosmotic stress damage and promote wound healing; in a mouse model of dry eye disease, they also promoted corneal repair and anti-inflammatory effects [291]; however, the mechanism of action remains unclear, and the animal studies involved small sample sizes, lacking direct comparison with standard clinical treatments.

#### 5.1.2. Systemic Administration

Intravenous administration is one of the primary routes for systemic delivery of PDNVs. Its advantages lie in high bioavailability and rapid systemic distribution, making it suitable for diseases requiring systemic treatment, such as cancer and infections. Following intravenous injection, PDNVs are distributed via the bloodstream and tend to accumulate in organs rich in the mononuclear phagocyte system (MPS), such as the liver, spleen and lungs [34]; this distribution pattern is closely related to particle size and surface properties. In contrast, distribution to the kidneys is typically low,likely constrained by particle size and the glomerular filtration barrier. The in vivo fate of PDNVs derived from different plant sources following intravenous injection varies significantly; for example, tea-derived nanovesicles can evade MPS capture and are partially distributed to the gut [286]; whereas nanovesicles derived from grapefruit are primarily captured by MPS organs such as the liver, lungs and spleen [286]. This may be related to differences in their lipid composition and surface charge; however, the distribution differences resulting from the specific plant species of origin require further investigation. Furthermore, some studies have reported that intravenous administration is associated with a certain degree of hepatotoxicity, nephrotoxicity and the risk of immune activation [286,292,293].

Compared with intravenous injection, oral administration offers more pronounced safety advantages. Chen et al. compared the in vivo behaviour of orally administered PDNVs with that of intravenously administered PDNVs, finding that intravenous administration triggered complement activation, an immune response and hepatic and renal toxicity, and that PEG surface modification was unable to fully mitigate these risks; whereas oral administration, due to the removal of immunogenic components from the vesicle surface by gastrointestinal enzymatic hydrolysis, resulted in significantly lower systemic toxicity than intravenous administration, and the remaining vesicles were still capable of exerting regulatory functions on distant organs [293]. Upon oral administration, certain types of PDNVs can resist gastrointestinal enzymatic degradation due to their nanocage structure [255]. This route is suitable for the treatment of gastrointestinal diseases, as the drug can act directly on the lesion and is more readily accepted by patients [188]. Multiple studies have confirmed that orally administered PDNVs can colonise and exert their effects in different regions of the intestine. For example, ginger-derived nanovesicles exhibit prolonged retention in the stomach, colon and upper ileum following oral administration, with preferential localisation in the intestine [119,294]; grapefruit-derived nanovesicles, on the other hand, accumulate in macrophages of the lamina propria of the caecum and colon, and can partially migrate to the liver [119,164]; grape-derived nanovesicles can penetrate the intestinal mucosal barrier, are taken up by intestinal stem cells, and activate the Wnt/β-catenin pathway, thereby promoting the proliferation and regeneration of the intestinal mucosal epithelium [7,119]. A growing body of evidence suggests that, following intestinal absorption, PDNVs can be distributed via the bloodstream to distant organs such as the liver, kidneys and brain. For example, orally administered lemon-derived nanovesicles are transported from the intestine to the kidneys, where they accumulate in renal tubular cells, thereby mitigating the progression of kidney stones [295]; pomegranate-derived nanovesicles, when taken orally, modulate the gut-liver axis and improve non-alcoholic fatty liver disease [296]; and following oral administration, garlic-derived nanovesicles act indirectly on the brain via the gut–brain axis, inhibiting high-fat diet-induced brain inflammation [297]. It should be noted that the currently more established method of brain-targeted drug delivery remains intranasal administration rather than oral administration. The advantages of oral administration lie in its non-invasive nature and high safety profile; however, the key to its success lies in overcoming the formidable gastrointestinal barrier [202].

Intraperitoneal injection, as one of the primary routes of administration for PDNVs, has been demonstrated in numerous studies [298,299]. Its advantages include rapid absorption, high bioavailability, and distribution primarily to immune organs (such as the spleen and lymph nodes) [286]; this characteristic confers unique value in immunomodulation and the treatment of intraperitoneal diseases. Furthermore, in studies on *Artemisia annua*-derived nanovesicles, Liu et al. compared intraperitoneal, intravenous, and intratumoural injection routes for their ability to deliver nanoparticles to subcutaneously implanted tumours and to control tumour growth. The results revealed comparable efficacy among these three routes [298], suggesting that for superficial solid tumours, both intratumoural (local) and systemic (intraperitoneal or intravenous) injection can achieve effective delivery [298]. Raimondo et al. isolated lemon-derived nanovesicles and compared the antitumour effects of intratumoural and intraperitoneal injection, finding that both routes significantly inhibited the growth of chronic myeloid leukemia xenografts, but intratumoural injection exhibited stronger activation of the TRAIL apoptosis pathway [278], indicating that different delivery routes may exert their effects through mechanisms of differing potency. However, similar to intravenous administration, intraperitoneal administration of PDNVs faces rapid clearance by the MPS and must therefore be ‘camouflaged’ through engineering modifications (such as PEGylation) to prolong circulation time [202].

The advantages of intramuscular injection lie in its rapid absorption, high bioavailability and complete avoidance of the first-pass effect [300]; it is suitable for drugs requiring rapid onset of action [301], those intended to bypass hepatic metabolism, or those formulated as long-acting preparations [302,303]. However, there is a potential risk of nerve and tissue damage [304]. Liu et al. administered isolated Goji berry-derived nanovesicles (GqDNVs) via intramuscular injection, directly into the quadriceps femoris muscle of mice with dexamethasone-induced muscle atrophy. The results showed that GqDNVs were localised to the muscle at the injection site without escaping to other tissues, significantly increasing the cross-sectional area of quadriceps muscle fibres and grip strength, thereby providing experimental evidence for the application of intramuscular PDNVs in the treatment of skeletal muscle diseases [258]. Toniolo, A et al. demonstrated that while intramuscular, oral, and intranasal administration of orange-derived nanovesicle mRNA vaccines all elicited serum IgM/IgG and neutralizing antibodies, intramuscular injection failed to induce mucosal IgA—a deficiency not shared by the oral and intranasal routes, which produced significant virus-specific secretory IgA [305]. Hence,for diseases requiring the blocking of pathogen infection at mucosal surfaces, intramuscular administration is at a clear disadvantage compared to the oral or intranasal route.

The selection of a delivery route for PDNVs is not an isolated decision but should be closely aligned with the physiological characteristics of the target organ, the physicochemical properties of the PDNVs themselves, and the anticipated therapeutic mechanism. It should be noted that engineering modifications can alter the biodistribution of PDNVs. Research into oral and transdermal administration of PDNVs is the most mature and widely applied; the former has entered the dietary supplement market, whilst the latter is already used in the cosmetics sector [306]; Although injection (intravenous, intraperitoneal, intramuscular) offers high bioavailability, its application is currently limited to animal models; intranasal administration is the fastest-growing approach for brain targeting, representing a non-invasive strategy for treating central nervous system disorders, though scaffold loading remains in the exploratory phase. Some research teams have claimed to have achieved administration via the reproductive tract, but there are currently no authoritative experimental reports to substantiate this. It is particularly important to emphasise that all evidence regarding the various routes of administration for PDNVs currently stems from in vitro experiments or animal models; they have not yet entered the stage of human clinical trials. Caution must be exercised when extrapolating these findings to humans, as in vitro experiments cannot simulate the complex physiological environment within the body, including the dynamic interactions of the immune system, clearance mechanisms within the bloodstream, and the long-term cumulative effects of metabolic by-products.

## 6. Clinical Translation and Commercial Exploration

At present, the clinical translation of engineered PDNVs requires overcoming four major bottlenecks: large-scale production, quality control, preservation techniques, and safety validation.

### 6.1. Scaling Up Production

Achieving high-purity, reproducible, large-scale production of natural PDNVs is a prerequisite for clinical translation. Process optimisation should focus on the coordinated regulation of upstream and downstream processing stages.

#### 6.1.1. Standardisation of Upstream Raw Materials

Variability in plant raw materials is a major cause of batch-to-batch inconsistency in PDNVs. During the upstream processing stage, attention should be paid to selecting the optimal plant parts based on variations in vesicle concentration and bioactivity across different plant tissues [96,307]. Furthermore, it is recommended to employ smart agricultural technologies to standardise the cultivation and harvesting of plant materials, ensuring consistency in growing environments and developmental stages, thereby guaranteeing the stability of vesicle yield and quality from the source. The use of artificial intervention to regulate cultivation conditions for targeted control of raw materials should be considered a key strategy within smart agriculture.

Taking the standardisation of upstream raw materials as an example, LED light regulation has demonstrated potential for enhancing EV yield at the laboratory scale. One study found that culturing edelweiss callus tissue under red LED light increased nanovesicle yield by 2.6-fold compared to dark conditions; this phenomenon has also been observed in centella asiatica and ginseng, suggesting that this strategy may be applicable across species [308]. However, this conclusion has not yet been replicated by independent laboratories, and scaling up the application faces multiple challenges. For instance, light penetration into callus tissue within bioreactors is limited, and uneven light intensity distribution may affect yield stability; furthermore, whether the functional advantages of nanovesicles obtained through LED treatment are maintained in in vivo models remains to be verified.

Beyond physical stimulation, the optimisation of culture methods is equally crucial. Suspension culture of plant cells utilises the characteristic of plant cells actively secreting nanovesicles into the culture medium, thereby avoiding the co-separation of intracellular debris during tissue disruption, and offering the potential to obtain PDNVs of higher purity [162]. However, this strategy currently remains at the laboratory proof-of-concept stage. The genetic stability of plant cell lines during long-term passaging remains unclear; efficient recovery processes for PDNVs from culture media have yet to be established; and the economic balance between cultivation costs and yield has not yet been assessed.

Another parallel culture method is the Temporary Immersion Bioreactor System (TIBS), which is suitable for the cultivation of tissue blocks such as callus. This system provides dynamic physical stimulation through computer-controlled periodic immersion and drainage, enabling efficient plant tissue proliferation and sustained secretion of PDNVs [309]. However, the isolation of nanovesicles from TIBS culture medium still relies on downstream processes such as ultracentrifugation (100,000× *g*), and the final particle size distribution and purity of the nanovesicles remain subject to the purification method; this technology is currently still in the preliminary exploratory stage.

Given the high growth rate and controllable cultivation characteristics of microalgae, this source is considered to have potential for further scaling up [55]. A study screened 18 microalgal species to identify seven EV-producing strains, including *Cyanophora paradoxa*, and confirmed their yield and particle size characteristics at a 50 mL laboratory scale [310]; building on this, another study focused on *Tetraselmis chuii* and used TFF to validate the feasibility of microalgal-derived nanovesicles production at a 7.5 L pilot scale, with yields comparable to those achieved by dUC [311]. However, the process stability and cost-effectiveness of these methods in large-scale photobioreactors (ranging from hundreds to thousands of litres) remain to be verified. LED-based plant cell culture systems offer the advantage of low energy consumption; strategies combining these with high-density culture modes are considered to have the potential to reduce production costs per unit yield [308], providing a possible approach for the scaled-up production of PDNVs, although this has not yet been empirically validated in the large-scale production of PDNVs. Precise control of culture conditions, such as temperature, light intensity and nitrogen nutrient levels, directly influences the yield, particle size distribution and protein content of nanovesicles [312]. Delivering PDNVs loaded with expression plasmids encoding CRISPR/Cas9 or its derivative tool dCas9-TEN into plant seeds, buds, or germ cells (e.g., pollen and ovules) holds the potential to generate heritable genetic variations, thereby accelerating the breeding of new crop varieties with desirable agronomic traits [39,313,314,315]. Furthermore, PDNVs can also be considered as siRNA carriers to enable transient regulation of gene circuits controlling flowering time, vernalisation requirements and drought tolerance [20,315], providing important tools for coping with extreme weather conditions under global climate change [20,315]. 

#### 6.1.2. Downstream Process Scale-Up

Although Section 2, ‘Isolation’, has detailed various technical platforms for the large-scale production of nanovesicles, it is important to note that there is a fundamental distinction between large-scale production and clinical translation or commercial application. The latter requires downstream processes to simultaneously meet four core requirements: high throughput, good manufacturing practice (GMP) compliance, process robustness and cost-effectiveness; however, most current research remains confined to the laboratory scale [55,169].

Current research into downstream processes for the large-scale production of PDNVs includes, firstly, membrane separation technologies, which are suitable for juice-derived sources such as fruit juices. One study utilised 750 kDa hollow-fibre membrane modules to process lemon juice and found that this process outperformed conventional methods in terms of impurity removal and nanovesicle recovery, demonstrating potential as a scalable alternative; however, it still faces challenges such as membrane fouling and the risk of shear forces damaging vesicle integrity [70]. Although the aforementioned technical approaches have demonstrated potential for scaling up, it remains unverified whether the reported yields can be replicated during scale-up, whether the functional integrity of the vesicles is compromised after scale-up, and whether the coefficient of variation between batches can be controlled. Furthermore, the matrix characteristics of PDNVs from different plant sources vary significantly; their large-scale separation methods cannot be simply replicated and must be specifically developed and optimised.

As a single technology struggles to balance factors such as yield and purity, the implementation of a combined strategy utilising multiple technologies is the core pathway to achieving large-scale production of PDNVs, a view that has become the consensus in numerous reviews. Researchers should focus on the combined use of multiple separation, concentration and purification technologies, utilising the complementary advantages of different methods to maximise separation efficiency [75]. Although existing studies have begun to explore large-scale production methods centred on TFF, combined with UC, UF, DF, AEX, Affinity Chromatography (AC) and others [55,316], these have largely focused on verifying technical feasibility rather than the optimisation of process parameters—specifically, how key process parameters (such as TFF shear rate, AEX elution conditions, and pH [317]) influence the yield, purity, and functional integrity of PDNVs, for which no definitive conclusions have yet been reached. Similarly, in the subsequent standardised drug loading process, optimal parameters must be established for loading methods such as electroporation, sonication and incubation to ensure consistency in loading efficiency and release kinetics across different batches. A deeper challenge lies in the fact that the specificity of plant-derived impurities (such as pectin and polyphenols) makes it impractical to directly apply mammalian quality control standards, whilst a unified definition of critical quality attributes for PDNVs remains lacking. The absence of assessment criteria for quantification (NTA, DGC), qualitative analysis (Western blot) and functional activity (cell uptake) also makes it difficult to avoid systematic errors [318]. Validation of safety and efficacy throughout the entire process is likewise a necessary prerequisite for clinical translation [319,320,321].

Regarding the large-scale production of PDNVs, Giancaterino et al. [55] and Staubach et al. [318], in their review, summarised existing separation technologies and noted that membrane separation processes (particularly TFF) outperform traditional methods in terms of impurity removal and nanovesicle recovery. These are considered the most promising technical pathways for process scale-up, capable of being used independently or in combination with chromatography techniques [318]. This assessment aligns with industrial experience accumulated in the fields of M-EVs and viral production, where technologies such as TFF and ion-exchange chromatography have been demonstrated to support automated operations and handle volumes ranging from hundreds to thousands of litres. However, the application of membrane separation technologies in PDNVs remains limited at present, primarily serving as auxiliary steps in conjunction with UC, and has not yet been validated as an independent large-scale platform [318]. Furthermore, translating the aforementioned technical routes into feasible schemes for the clinical-scale production of PDNVs will still require systematic process development and validation tailored to the specific characteristics of different plant matrices (such as pectin and polyphenol interference).

### 6.2. Quality Control

In the quality control of PDNVs, in addition to the commonly used analytical techniques mentioned in Section 2.2 Characterisation—such as TEM/SEM, cryo-EM, NTA, DLS, Fluorescence-Activated Cell Sorting (FACS) and CE—high-performance liquid chromatography (HPLC) and nano-plasma sensing (NPS) are also key analytical techniques.

Compared with the other two categories of techniques, the advantage of HPLC lies in the accurate quantitative analysis of active components, and the method is well-established and easy to standardise; however, its limitation is that it cannot distinguish between free and encapsulated drugs [322]. Taking ginger-derived nanovesicles as an example, Man et al. used HPLC to quantitatively analyse the differences in the content of 6-gingerol (6 G), 8-gingerol (8 G) and 10-gingerol (10 G) between nanovesicles and sliced ginger. The results showed that, on a dry weight basis, the content of the three gingerols in nanovesicles was 10.21–32.36 times that of ginger slices, and the higher the lipophilicity (Log *p* value), the stronger the enrichment capacity (10 G > 8 G > 6 G) [323]. This analytical approach can be applied to the quality control of active ingredients in other PDNVs, and subsequent integration with mass spectrometry can further enhance detection specificity. Several studies published in recent years indicate that HPLC is becoming an important tool for the quality control and drug loading evaluation of PDNVs [322,323].

Compared to HPLC, the advantage of CE lies in its ability to simultaneously detect both free drug and drug encapsulated within PDNVs, thereby assessing formulation stability and preventing drug leakage [187], demonstrating considerable potential in the quality control of PDNVs. However, its detection throughput is relatively low, and it is sensitive to sample matrices; high-abundance impurities may interfere with separation [324]. For example, following the loading of DOX onto lemon-derived nanovesicles, researchers were able to directly and sensitively monitor drug loading efficiency and assess formulation stability in real time using CE technology, without the need for prior removal of free DOX or complex sample pretreatment. This avoided drug adsorption losses and operational complexity associated with traditional methods such as UC, dialysis or filtration [187]. Currently, this technique has only been applied to nanovesicles from two species of the genus Citrus (*Citrus limon* and *Citrus aurantifolia*) [325], and its universality has not been widely validated. However, studies have shown that the fluorescence intensity of DOX is affected by pH and microenvironmental quenching effects, which may compromise the quantitative accuracy of CE detection [326]. To address this issue, future research could consider employing ratiometric fluorescence or dual-fluorophore ratiometric fluorescence [327] to correct the aforementioned interferences; however, the feasibility of these methods remains to be validated.

Compared with HPLC and CE, the unique advantage of NPS lies in its ability to monitor interactions at the solid–liquid interface in real time. It is suitable for detecting drug accumulation in the PDNV interface region, and is particularly well-suited for high-sensitivity analysis of low-concentration samples. Currently, the application of NPS in the quality control of engineered PDNVs remains in the exploratory stage, with only a few studies reported. For example, the drug distribution in lemon-derived nanovesicles loaded with DOX can be verified using NPS technology; however, this method is currently used primarily for qualitative analysis and cannot accurately quantify the drug [187], and the detection results are susceptible to the condition of the sensor surface.

### 6.3. Preservation

The choice of preservation technique for PDNVs must be determined by taking into account the duration of storage,the plant origins, the intended application, and the key active ingredients (Table 1, Supplemental Table S4).

The selection of PDNVs preservation strategies should be based on plant source [328], storage duration, application scenario, and core bioactive components. Refrigeration at 4 °C is suitable for temporary storage or transportation (within days), largely preserving vesicle integrity but requiring avoidance of prolonged storage to prevent activity loss [328,329]. For medium-term storage (weeks to months), freezing at −20 °C with aliquoting is recommended to avoid repeated freeze-thaw cycles, which significantly compromise PDNVs stability [328,329]. For long-term storage (over six months), −80 °C cryopreservation is advised, ideally with liquid nitrogen flash-freezing as a pre-treatment [328].

Lyophilization is the core technology for long-term room-temperature storage; lyophilized PDNVs remain stable at room temperature for 60 days without significant changes in particle size, morphology, or zeta potential [29]. The key to successful lyophilization is the formulation of protective agents, such as cryoprotectants (e.g., trehalose, sucrose, mannitol) or antioxidants [330]. The patented formulation “trehalose + low-molecular-weight hyaluronic acid” (US20230285302A1, AVECIN-Biopharma Oy, 2023) [331] has been shown to preserve apple-derived nanovesicles at room temperature for at least 6 months [331], offering a basis for further optimization according to plant source characteristics. Notably, differences in membrane lipid composition, surface charge, and bioactive cargo among vesicles from different sources necessitate source-specific optimization of pre-lyophilization pH, freezing rate, and sublimation temperature [331,332].

For formulation-based stabilization, chemical protectants such as PBS, Saliguard^®^ TMO, and 1,3-butylene glycol are commonly used to enhance the efficacy of refrigerated or frozen storage [329]. For instance, Kim et al. reported that *Dendropanax morbifera* leaf-derived nanovesicles (LEVs) combined with TMO and stored at 4 °C exhibited the best stability among tested conditions [329]. PBS supplemented with human albumin and trehalose (PBS-HAT) has been shown to significantly improve the cryostability and recovery of extracellular vesicles [330]. In addition, Pickering emulsion-assisted stabilization technology employs natural microcrystalline cellulose and xanthan gum to form a three-dimensional network via hydrogen bonding and electrostatic interactions, encapsulating multiple PDNVs and avoiding the vesicle damage caused by conventional emulsifiers. This system maintains intact bilayer vesicle structures for at least 3 months under conditions ranging from −18 °C to 50 °C, as well as under light exposure and mechanical agitation, as validated by accelerated testing (45 °C, 3 months) [333], providing a key technological foundation for the application of PDNVs in cosmetics and complex liquid formulations.

### 6.4. Clinical Trials and Translational Challenges

Research into PDNVs has shifted from exploring fundamental mechanisms to preclinical therapeutic validation. As shown in Table 2, clinical trials for PDNVs remain, on the whole, at a very early exploratory stage. Among the six registered trials, most are in Phase I or exploratory stages, one has been withdrawn, and no Phase II or III trials have been reported. The two completed trials (NCT04879810, NCT01668849) have not published their results, limiting evidence accumulation. Furthermore, most studies lack clear guidance on the dosing strategy for PDNVs [52,55]; they only provide dosage information for the encapsulated drug or the total mass of the extract, without specifying quantitative indicators of the vesicle carrier itself (e.g., particle number, protein content, or encapsulation efficiency). These indicators are critical for establishing the safety and therapeutic profile of PDNVs. Moreover, some studies use whole plant fruits or crude extracts as the intervention, which deviates from rigorous plant-derived vesicle-based research. In terms of indications, the six studies cover six distinct disease categories—colon cancer, inflammatory bowel disease (IBD), cardiometabolic disorders, polycystic ovary syndrome (PCOS), oral mucositis, and gut microbiota dysbiosis. This dispersion of research focus means that no single indication has accumulated sufficient clinical evidence. Furthermore, only a few plant sources (e.g., ginger, grape, lemon) have entered clinical trials, leaving many promising sources still at the laboratory stage. Together, these shortcomings indicate that PDNVs remain far from clinical translation.

### 6.5. Regulatory Pathways and Commercial Applications

#### 6.5.1. Regulatory Pathways for Pharmaceutical Products

When PDNVs are developed as medicinal products, there are fundamental differences in their regulatory classification across the US, Europe and China. The US Food and Drug Administration (FDA) tends to classify them as biologics, regulated by the Center for Biologics Evaluation and Research (CBER) [336], requiring submission through the Biologics License Application (BLA) pathway. This process mandates the definition of the active ingredient and completion of rigorous chemical, manufacturing, and control (CMC) evaluations, followed by clinical trials demonstrating safety and efficacy before marketing approval can be sought. The European Medicines Agency (EMA), on the other hand, may classify them as Advanced Therapy Medicinal Products (ATMPs), requiring rigorous evaluation of their pharmacological properties, manufacturing processes and quality control by the Committee for Advanced Therapies. Furthermore, if nanoparticles constitute more than 50% of the product, the substance is subject to additional nanospecific data requirements under the Registration, Evaluation, Authorisation and Restriction of Chemicals (REACH) Regulation, including more detailed physicochemical characterisation and toxicological assessment. In June 2025, China’s National Medical Products Administration (NMPA) for the first time explicitly incorporated EVs into the regulatory framework for advanced therapy medicinal products (ATMPs), placing them on par with stem cell and gene-edited products, and mandating compliance with Good Laboratory Practice (GLP), Good Clinical Practice (GCP), and GMP standards.

#### 6.5.2. Commercial Products

In contrast to the stringent regulation in the pharmaceutical sector, the commercialisation of PDNVs in non-pharmaceutical fields has progressed relatively faster, forming a development pattern where “cosmetics lead and pharmaceuticals follow”. Regarding plant sources, grapes, due to their high polyphenol content, have become one of the most widely used sources internationally; green tea and ginseng, owing to their antioxidant and anti-inflammatory properties, have seen relatively mature applications in the cosmetics sector; whilst medicinal and edible plants such as ginger and *Aloe vera* are attracting attention in medical research; distinctive plants such as sargassum, olive leaves and microalgae have also become key areas for differentiated market positioning. Currently, some products have completed initial commercial validation in the cosmetics sectors. In terms of regulatory requirements, the US FDA stipulates that cosmetics must demonstrate safety but do not require approval; however, if therapeutic effects are claimed, they must be reclassified as over-the-counter medicines for registration; Europe regulates under the Regulation (EC) No 1223/2009 of the European Parliament and of the Council of 30 November 2009 on cosmetic products, requiring comprehensive safety assessments and ingredient reporting, with market access requirements being strict. China maintains relatively strict oversight of exosome-derived ingredients in cosmetics; the ‘Catalogue of Cosmetic Ingredients in Use’ does not include any ‘exosome’-related ingredients, and human-derived exosomes are explicitly prohibited, though no explicit restrictions have yet been imposed on PDNVs.

#### 6.5.3. Current Bottlenecks and Recommendations

The key bottlenecks currently hindering the commercialisation of PDNVs are primarily evident in three areas: firstly, a lack of uniform classification standards, with the US, EU and China categorising them as biologics (US), advanced therapy medicinal products (EU) and cell therapy product under therapeutic biological products (China) respectively, requiring multinational companies to navigate multiple regulatory requirements; secondly, an incomplete evaluation framework, as no unified standards have yet been established for assessment methods concerning pharmacokinetic characteristics and long-term immunogenicity risks; thirdly, the absence of industrialisation standards, with large-scale production facing technical bottlenecks such as batch consistency and quality control. This paper recommends promoting international coordination to jointly establish a unified definition and classification principles for PDNVs with regulatory authorities in Europe and the US; simultaneously, a tiered evaluation system should be established, with the pharmaceutical sector strictly adhering to GLP/GCP/GMP and formulating targeted guidelines; consideration should also be given to building and refining industrialisation standards based on GMP production lines, clarifying extraction processes, quality control indicators and batch consistency requirements; Finally, international mutual recognition should be strengthened by promoting cross-border acceptance of drug master file documentation and ATMP review data among the FDA, EMA and China’s NMPA, thereby accelerating the global industrialisation process.

## 7. Conclusions and Outlook

The review traces the nearly six-decade history of research into PDNVs, providing a comprehensive overview ranging from their biogenesis, isolation and purification, and component identification to their engineering and clinical translation. Although PDNVs demonstrate unique advantages in the fields of nanomedicine, skincare and nutritional health due to their natural origin, low immunogenicity and cross-species communication capabilities, this review reveals that the field is currently at a critical stage of transition from experimental research to clinical application, with numerous core bottlenecks yet to be overcome.

At the level of isolation and purification, a single technology is no longer capable of balancing multiple factors such as yield, purity and cost; the integration of multiple technologies has become the only viable path to large-scale production. However, the standardisation of process parameters for different plant matrices remains virtually non-existent, directly leading to batch-to-batch heterogeneity becoming the primary obstacle to clinical translation. In terms of component characterisation, although plant-specific markers such as PEN1, TET8, DGDG and MGDG have been identified, the field still lacks standardised surface markers validated across multiple species on a large scale, which limits the standardisation of separation protocols and the in-depth analysis of mechanisms of action.

Engineered modification has shifted from single-function conferral to multimodal synergy. Through the combined application of chemical conjugation, genetic engineering and functional carrier composite systems, PDNVs have achieved significant improvements in targeting, circulation half-life and drug-loading profiles. However, their clinical translation still faces three mechanistic challenges. Firstly, the drug-loading mechanism remains unclear; there is a lack of systematic research into the impact of existing physical or chemical loading methods on vesicle membrane structural integrity and intracellular transport pathways, and PDNVs from different plant sources exhibit significant variations in their response to the same drug-loading strategy. Secondly, in vivo behaviour is difficult to predict. PDNVs are rapidly cleared from the bloodstream, and the in vivo distribution characteristics of nanovesicles from different sources vary significantly; however, there is currently a lack of cross-species pharmacokinetic models to accurately predict their fate in the human body. Thirdly, safety data are insufficient; the risk of immunogenicity under conditions of long-term repeated dosing has not yet been fully assessed through standardised non-clinical studies, and the potential toxicity associated with the accumulation of PDNVs in organs such as the liver and spleen following intravenous injection still requires further verification.

A hierarchical system has been established for PDNVs preservation technologies, in which 4 °C refrigeration is suitable for short-term handling, −20 °C and −80 °C freezing cover medium- to long-term storage needs, and lyophilization together with Pickering emulsion technology provides feasible solutions for long-term room-temperature storage. However, it should be noted that strategy selection must comprehensively consider plant source, storage duration, application scenario, and core bioactive components. Single-particle multiparameter analysis elevates quality control from averaged metrics to a new level of heterogeneity characterization. However, these techniques have yet to establish industry-wide standards, directly resulting in poor data comparability across different studies.

The selection of administration routes is shifting from empirical trial and error towards mechanism-driven, precise matching. The oral route demonstrates distinct advantages in the treatment of metabolic diseases and intestinal inflammation, owing to PDNVs’ unique gastrointestinal tolerability and their potential for delivery via the gut–organ axis; intranasal administration, meanwhile, offers a differentiated route for delivering treatments to the central nervous system due to its ability to cross the blood–brain barrier. Although intravenous injection enables rapid systemic distribution, the accumulation effects in the liver and spleen, as well as the potential risk of immune activation, cannot be overlooked.

In terms of clinical translation, clinical trials involving PDNVs remain at an early exploratory stage, with no product having yet completed Phase III clinical trials or received marketing authorisation [55,335]. The current key bottlenecks are concentrated in three areas: firstly, the lack of batch-to-batch consistency standards in large-scale production; secondly, the risk of immunogenicity in scenarios involving long-term repeated administration has not yet been fully assessed through standardised non-clinical studies; and thirdly, differences in regulatory frameworks across regions increase the complexity of cross-border development. Experience in advancing M-nanovesicles to clinical application may serve as a reference for PDNVs; however, fundamental differences between the two in terms of biological mechanisms, biomarker systems and quality control standards necessitate the establishment of independent translational pathways and evaluation systems for PDNVs. To bridge the gap between preclinical research and human application, this paper suggests that future research should focus on improving existing model systems. In vitro models could shift from 2D monolayer cultures to 3D organoids and microfluidic chips, expanding from single cell lines to co-cultures of immune cells, whilst simulating the hypoxic and acidic conditions found in vivo. At the animal model level, the introduction of physiologically-based pharmacokinetic (PBPK) models, combined with AI algorithms to optimise parameters, can predict dose–response variations in virtual patient populations. By establishing more accurate in vitro–in vivo correlation models, it is hoped that the reliability of extrapolating preclinical data to humans can be enhanced.

In the future, the focus will no longer be on local optimisation of individual technologies, but rather on the depth of interdisciplinary integration and the breadth of systematic thinking.

Research into TCM-derived vesicles, represented by Chinese Herbal Medicine-derived Extracellular Vesicle-like Particles (CHM-EVLPs), provides a new technical pathway for elucidating the active substance basis of TCM decoctions [337]. By isolating and identifying the active components of TCM decoctions within vesicles, this approach not only effectively avoids the destruction of heat-sensitive biomolecules caused by traditional processing methods such as high temperatures and alcohol, but also endows the active components with unprecedented stability and bioavailability through the phospholipid bilayer structure [338]. Future research should focus on establishing standardised nomenclature rules and quality control systems for CHM-EVLPs, as well as identifying their key biomarkers, to promote the standardised application of TCM-derived vesicles in the field of nanomedicine [337,339].

The application of machine learning and deep learning technologies in PDNV research is still in its infancy; currently, only a small number of studies have utilised large language models for the intelligent mining of PDNVs’ active components [340]. Deeper applications of AI in areas such as separation process optimisation [341,342], subpopulation classification [341,342], prediction of molecular interaction networks [341], and quality control [178,341,342,343,344,345] remain at the theoretical conceptual stage and have yet to be experimentally validated. The Gramord platform utilises deep learning to identify co-assembly compatibility between small molecules [346]. If such computational strategies were transferred to the PDNV system, they could potentially be used in the future to predict drug combinations suitable for co-loading; however, this approach currently remains an extension at the methodological level, and its feasibility within the PDNV field has yet to be confirmed.

The development of smart, stimulus-responsive PDNV carriers is emerging as a frontier in precision drug delivery. The design of smart drug delivery systems capable of responding to tumour microenvironment pH, redox levels or enzymatic signals, or the implementation of combination therapy strategies—such as loading PDNVs with chemotherapeutic agents, co-administering immune checkpoint inhibitors, or constructing hydrogel composite systems—holds promise for achieving precise drug release and controlled activation. This could provide new pathways for overcoming delivery barriers in solid tumours and overcoming drug resistance [243,244,246,247,248,249,347].

PDNVs represent a paradigm shift in the biomedical field, offering novel approaches by integrating nanomedicine, skincare and nutritional health. Their natural origin, low immunogenicity and drug delivery potential bring new hope to patients suffering from diseases for which effective therapies have long been lacking. However, the transition from the laboratory to clinical practice still requires overcoming multiple obstacles that remain, including large-scale production, standardised quality control, long-term safety validation and regulatory coordination.

## Figures and Tables

**Table 1:** The Selection of PDNV Preservation Technology.

Saving Method	Advantages	Limitation
Refrigeration at 4 °C	Easy to operate, suitable for short-term experiments	Prone to protein degradation and loss of small particles
−20 °C Freezing	No need for cryogenic equipment	Prone to aggregation, severe damage from repeated freeze–thaw cycles
−80 °C Cryogenic Freezing	Best long-term stability, minimal activity loss	Relies on specialized equipment, high transportation cost
Lyophilization (with protectant)	No cold chain required, low transportation cost, high reconstitution activity	Requires optimization of protectant formulation, complex process
Storage Solution Optimization	Simple operation; utilizes PBS/Tris-HCl/ectoine to build stable system	Requires low-temperature handling; avoid freeze–thaw cycles; needs compatible protectant screening
Pickering Emulsion	Resists temperature/light/agitation fluctuations; avoids traditional emulsifier damage; maintains vesicle integrity	Complex technology; relies on natural microcrystalline cellulose and xanthan gum network

**Table 2:** Overview of Clinical Trials Related to PDNVs.

Research Object	Research Content	Research Stage	ClinicalTrials.gov Identifier	Reference
Curcumin-loaded exosomes	Colon cancer treatment	Recruiting	NCT01294072	[334]
ginger exosomes, curcumin, curcumin-loaded ginger exosomes	IBD ginger exosomes/curcumin trial	Completed, results not yet announced	NCT04879810	[55]
citrus lemon	Metabolic Syndrome treatment	results not yet announced	NCT04698447	[55]
Ginger exosomes, Aloe exosomes	PCOS treatment	Withdrawn	NCT03493984	[335]
grape exosomes	Treatment of chemo-radiation-induced oral mucositis in head and neck cancer patients	Completed, results not yet announced	NCT01668849	[55]
*Rubus chamaemorus* exosome, *Vaccinium vitis-idaea* exosome	Effects on Gut Microbiota and Immune Status	currently underway	NCT07381933	/

This table adopts a large amount of information from reference [335] and https://clinicaltrials.gov.

## Data Availability

No new data were created or analyzed in this study. Data sharing is not applicable to this article.

## Supplementary Materials

The following supporting information can be downloaded at: https://www.mdpi.com/article/10.3390/biom16050705/s1, Table S1: Comparison of Major Separation Methods; Table S2: Features, Biological Functions, and Mechanism of Action of PDNV; Table S3: Applications of PDNV in the treatment of various diseases. Table S4: Preservation methods for different PDNVs. References [54,140,152,348,349,350,351,352,353,354,355,356,357,358,359,360,361,362,363,364,365,366,367,368,369,370,371,372,373,374,375,376,377,378,379,380,381,382,383,384,385,386,387,388,389,390,391,392,393,394,395,396,397,398,399,400,401,402,403,404,405,406,407,408,409,410,411,412,413,414,415,416,417,418,419,420,421,422,423,424,425,426,427,428,429,430,431,432,433,434,435,436,437,438,439,440,441,442,443,444,445,446,447,448,449,450,451,452,453,454,455,456,457,458,459,460,461,462,463,464,465,466,467,468,469,470,471,472,473,474,475,476,477,478,479,480,481,482] are cited in the supplementary materials.
